# Dynamical analysis of the SVEIR-M epidemic model with age structure under media coverage^☆^

**DOI:** 10.1016/j.idm.2025.11.004

**Published:** 2025-11-15

**Authors:** Jianrong Wang, Xue Yan, Xinghua Chang, Maoxing Liu

**Affiliations:** a School of Automation and Software Engineering, Shanxi University, Taiyuan, 030006, PR China; b School of Mathematics and Statistics, Shanxi University, Taiyuan, 030006, PR China; c Department of Mathematics, Taiyuan University, Taiyuan, 030000, PR China; d School of Science, Beijing University of Civil Engineering and Architecture, Beijing, 102627, PR China

**Keywords:** Media coverage, Stability analysis, Age structure, Uniform persistence

## Abstract

With the frequent emergence and spread of new infectious diseases, poses severe threats to public health, and the government often relies on non-pharmaceutical interventions to cope. Meanwhile, the impact of media information on public behavior and health awareness is increasingly significant, becoming an indispensable factor in epidemic prevention and control. This paper constructs an SVEIR-M infectious disease model integrating age structure and media coverage mechanisms, depicting the differences in individuals’ acceptance of media information and the effectiveness of vaccination at different age stages. The model introduces complex factors such as immune waning, latent development age, and media information dissemination, and systematically analyzes the existence and stability of disease-free and endemic equilibrium points using partial differential equations and Volterra integral tools. It is proved that the basic reproduction number *R*_0_ plays a threshold role in characterizing the dynamical properties of the system, and the global stability of equilibrium points under different conditions is demonstrated by constructing Lyapunov functions. In addition, the uniform persistence of the system is analyzed, and the correctness of the theoretical analysis is verified through numerical simulations, discussing the impact of different intervention measures on epidemic development. The research results show that media publicity and vaccination can significantly reduce the infection and mortality rates, and their combination can more effectively control the spread of the epidemic.

## 1 Introduction

Emerging infectious diseases and those lacking effective treatment options not only pose a threat to public health but also provoke widespread panic, significantly impeding social development and stability. Following an epidemic outbreak, governments typically implement non-pharmaceutical interventions, such as lockdowns, mass testing, and home quarantine, to curb the spread of infectious diseases on a large scale. However, these measures also introduce numerous disruptions to the normal functioning of society and exert a substantial impact on social and economic progress. Nonetheless, the role of media information reporting and publicity as an auxiliary tool should not be overlooked. During infectious disease outbreaks, a vast array of media reports and publicity efforts profoundly influence public awareness and behavior, empowering individuals to comprehend the disease accurately, bolstering residents’ self-protection capabilities, and guiding them toward adopting safe and healthy lifestyles and behaviors. Traditional mainstream media, including television news, traffic radio, and official websites, have long served as the primary sources of information for the public. However, with the rapid development of social platforms and short videos, the speed of information dissemination is faster, the scope is wider, and the push is more accurate, increasing the efficiency of the public in obtaining various kinds of information (Roy et al., 2020). The research direction of behavioral epidemiological models based on information dissemination and impact is diverse and fruitful, but it is still in the initial exploration stage. Issues such as how to collect disease information, how to quantify it accurately, how to couple human behavior factors, and how network topology affects information dissemination are all in urgent need of in-depth discussion and research.

Age is an important label for characterizing individual heterogeneity. Physiological age can well reflect a person's physical function in defending against diseases and also reflect an individual's social contact structure, which creates differences in the probability of an individual being infected and the ability to infect others. The strength of infectiousness is not only related to an individual's physiological age but also to the length of time since infection (infectious age). Moreover, the strength of susceptibility is also related to the duration of immunity after vaccination (immune age). The heterogeneity of infectiousness among individuals of different ages and the heterogeneity of infectiousness among individuals at different stages of infection are profoundly affecting the spread of infectious diseases. For example, according to the COVID-19 epidemic statistics from the US CDC on October 4, 2020, more than 50 % of the infected population in the United States were aged 18–50, with a total mortality rate of 2.8 %, and the mortality rate for those aged 18–50 was over 3 %. Although individuals aged 60 and above have a higher mortality rate, their infection rate is not high. In addition, there are also significant differences among individuals of different age groups in terms of the channels and cognition for receiving media information.

Therefore, targeting the differences in the spread of emerging infectious diseases and the diversity in media information cognition among individuals with different age structures, integrating age structure, information time delay, and awareness behavior into infectious disease modeling under the influence of media information can more finely and accurately depict the co-evolutionary process of individual disease awareness and infectious diseases in reality. Analyzing the dynamical behavior of the coupled system model and exploring the optimal control strategies for the coupled system will have strong practical significance and theoretical reference value.

With the development of mobile networks and information technology, the speed and scope of information dissemination in social networks have been greatly enhanced, and there have also been many fruitful developments in behavioral epidemiology. Once people become aware of the hazards and transmission characteristics of infectious diseases, they usually change their daily behaviors and take preventive measures to reduce the risk of infection. Currently, there is very limited theoretical research on how social media and human behavior jointly affect the spread of infectious diseases in the host population. In recent years, some research achievements on coupling disease information have promoted the rapid development of infectious disease behavioral dynamics modeling. Du et al. (Du et al., 2021) coupled the dynamics model of individual awareness of epidemic risk, the behavioral adoption model of disease prevention, and the SEIR epidemiological model in one framework to comprehensively evaluate the interactions of various influencing factors. Buonomo et al. (Bentout et al., 2021) considered an infectious disease model without mandatory COVID-19 vaccination. Considering individuals' hesitation to get vaccinated, they used a media information function with information time delay to describe the amount of information. Under extensive information coverage and short time delay, vaccine hesitation and refusal are better controlled. Li et al. (Li & Xiao, 2022) used a double non-linear function to investigate the impact of saturated media reporting and limited medical resources on disease transmission. Huo et al. (Huo & Yu, 2023) constructed a coupled negative information behavioral epidemiological dynamics model, introducing the Heaviside step function to explore the impact of the decision-making adoption process on the evolution of each layer. The study shows that increasing the publicity efforts of mass media and enhancing individuals’ self-awareness can help control the epidemic. Li et al. (Li et al., 2018) studied an SI epidemic model with feedback control in a patchy environment, showing that under appropriate controllers, the endemic equilibrium point can be controlled at a lower level and still remain stable. The research results of infectious disease behavioral dynamics are abundant. The following mainly analyzes the current situation of two hot issues: information time delay and age structure under the influence of media information.

The infection risk of infectious diseases varies with age, and vaccination programs are subject to the intervention of information dissemination. The age structure of the host population is a key factor in the spread and control of infectious diseases. For example, this is more evident in childhood infectious diseases such as chickenpox, hand-foot-mouth disease, pertussis, and norovirus. The natural mortality rate of individuals, the progression rate from incubation to infection, the recovery rate, and the vaccination rate are all related to the age of the population. Kuniya et al. (Kuniya, 2019) studied the appearance of periodic solutions through Hopf bifurcation in an age-structured SIR epidemic model, taking specific age as a bifurcation parameter and obtaining sufficient conditions for the occurrence of Hopf bifurcation. Li et al. (Li et al., 2019) studied a stochastic age-structured SIR model and proved the existence and uniqueness of global positive solutions using semigroup theory. Bentout et al. (Bentout et al., 2021) considered the high mortality rate of COVID-19 among the elderly and the sick, and established an age-structured model to predict the peak time and number of infected cases before and after the implementation of non-pharmaceutical interventions. Huang et al. (Huang et al., 2022) considered an SEIR-type age-structured epidemic model with vaccination and standard incidence rate. The study shows that if the transmission rate cannot be further reduced, the vaccination rate needs to be maximized, and vice versa. Khatua et al. (Khatua & Kar, 2020) proposed an epidemic model with stage structure, incorporating media awareness and assuming that the disease can only spread among the mature population. The study found that the threshold parameter is independent of any parameter related to media awareness, but the awareness parameter can effectively control the disease. Sun et al. (Sun et al., 2023) studied an age-structured SVIR epidemic model with two delays in vaccination and latency. In this model, one delay represents the latency period of the disease, while the other delay represents the minimum infection age at which infected individuals become infectious. The model has very complex dynamical behavior, and the existence of stability switches is proved. Sonveaux et al. (Sonveaux & Joseph, 2022) studied an age-dependent epidemic model and designed a state feedback vaccination law to eradicate the disease, providing a linearized state feedback vaccination law and obtaining conditions to ensure the stability of the closed-loop system and the positivity of the feedback control. People in different age structure stages have different infectivity for specific diseases, and the amount of media information also has a certain impact on different age groups of susceptible populations. In the existing literature, there are few studies on the impact of media information on age-structured epidemic models for specific diseases. Therefore, exploring such issues is challenging.

A growing literature investigates how mass media or information campaigns alter epidemic dynamics via awareness-driven contact reduction, vaccination uptake, or optimal control. Foundational work on influenza shows that media coverage can change transmission and even interact with control costs and pulse vaccination strategies (Tchuenche et al., 2011). Media functions that decrease effective contact rates—often modeled as exponential or Holling-type responses—have been analyzed with threshold conditions and stability properties (Tchuenche & Bauch, 2012). More recently, comparative studies demonstrate that different media functions can substantially shift peak timing, final size, and sensitivity indices in SIR-type models (Fome et al., 2023). Media has also been embedded in optimal-control frameworks together with treatment or vaccination to quantify policy trade-offs (Diagne et al., 2024). For HIV specifically, recent modeling links media campaigns to changes in the basic reproduction number, bifurcation behavior, and parameter sensitivities (Omondi et al., 2024). Meng et al. (Meng et al., 2025) investigated an HIV model with continuous infection-age structure and nonlinear incidence rates. By employing the method of characteristics and constructing auxiliary functions, they proved the existence of a global attractor for this type of model. This implies that the long-term behavior of the system will converge to a compact attracting set, providing a solid foundation for analyzing the long-term dynamics under the coupling of complex age structure and nonlinear transmission. Luo et al. (Luo et al., 2025) constructed a multi-group SVIR model with nonlocal diffusion, incorporating media-related nonlinear incidence rates and spatial heterogeneity. The results showed that the nonlocal diffusion kernel and the treatment rate are key factors in determining the epidemic outcome. Although media coverage does not change the ultimate outcome, it can significantly suppress the intensity of transmission and the density of infections. Hao et al. (Hao et al., 2025) proposed an SEIRSHM model with general birth rates, media coverage, and limited medical resources. Their work demonstrated the conditions for disease extinction, characterized the existence of endemic equilibria, and illustrated various complex dynamical phenomena. It further indicated that media information and constraints on medical resources have significant impacts on the epidemic process.

Unlike these studies, our work integrates a continuous age structure for exposure and vaccination into a media-aware SVEIR-M framework, and establishes existence of equilibria, local/global stability, and uniform persistence. This fills a gap where media effects and age heterogeneity jointly shape transmission and control outcomes.

In this paper, an age-structured SVEIR-M epidemic model under the influence of media is proposed. Unlike previous studies, the impact of media reporting on the transmission dynamics of COVID-19 is comprehensively considered, and a detailed theoretical analysis of the model is carried out. Section 2 constructs the age-structured SVEIR-M epidemic model. Section 3 proves the existence of equilibrium points. Section 4 proves the local stability of steady-state solutions, the uniform persistence of the model, and the global stability of steady-state solutions. Section 5 verifies the above theoretical results through numerical simulation. Section 6 presents a summary evaluation and prospects for future research.

## 2 Model construction

**Fig. 1:**
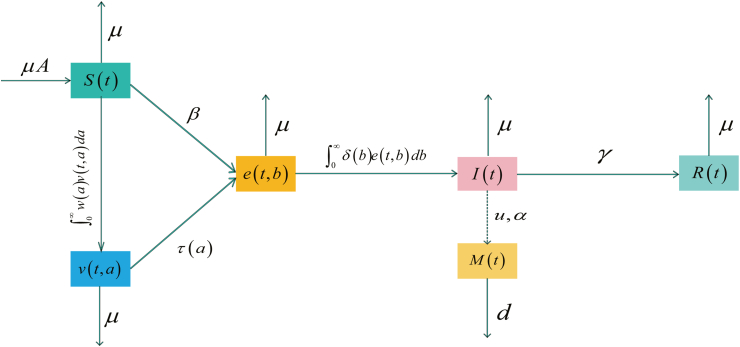
The flow chart.

Based on existing research results, this paper constructs an SVEIR-M epidemic model that takes into account the influence of media and age structure. The model divides the total population into susceptible individuals, vaccinated individuals, exposed individuals, infected individuals, and recovered individuals, and introduces a media information variable to describe the enhancing effect of media reporting on public awareness of protection. Specifically, the susceptible individuals *S*(*t*) represent those who are not infected and have not been vaccinated. The vaccinated individuals *V*(*t*) are described by the function *v*(*t*, *a*) according to the vaccination age, with the total number being $V\left(t\right)=\int_{0}^{\infty }v\left(t,a\right)\;da$; the exposed individuals *E*(*t*) are represented by the density function *e*(*t*, *b*), satisfying $E\left(t\right)=\int_{0}^{\infty }e\left(t,b\right)\;db$; the infected individuals *I*(*t*) and the recovered individuals *R*(*t*) represent those in the infectious period and those who have recovered, respectively. The media information variable *M*(*t*) reflects the cumulative effect of media reports and publicity in the system and exerts a negative feedback effect by adjusting the infection rate of the susceptible individuals. In the absence of vaccination, newborns enter the susceptible population at a constant rate of *μA*, where *μ* is the natural mortality rate. Due to the impact of media on the epidemic, it is assumed that the infection rate of susceptible individuals is affected by the cumulative awareness of media reports *M*(*t*). Let *v*(*t*, *a*) denote the density of vaccinated individuals of age *a* at time *t*, where *a* represents the vaccination age, and $V\left(t\right)=\int_{0}^{\infty }v\left(t,a\right)\;da$ represents the total density of vaccinated individuals. The rate of waning immunity acquired through vaccination is given by *w*(*a*), hence the rate at which vaccinated individuals return to the susceptible category is $\int_{0}^{\infty }w\left(a\right)v\left(t,a\right)\;da$. Susceptible and vaccinated individuals, once in contact with infected individuals, become new individuals. They enter the stage of being infected with the disease but the host is not yet infectious, a stage known as the latent period. It is assumed that both susceptible and vaccinated individuals enter the latent class, the density of which is denoted by *e*(*t*, *b*), where *b* is the duration spent in the latent period, referred to as the latent development age. $E\left(t\right)=\int_{0}^{\infty }e\left(t,b\right)\;db$ represents the total density of exposed individuals. The removal rate of exposed individuals is given by the function *δ*(*b*). Therefore, the total rate at which individuals in the latent class enter the infected class alive is $\int_{0}^{\infty }\delta \left(b\right)e\left(t,b\right)\;db$. The contact rate *β*(*M*(*t*)) describes the negative feedback of susceptible individuals on the spread of infectious diseases and is a decreasing function of *M*(*t*). The flow chart of the model is shown in Fig. 1.

The model describing the evolution of these state variables can be described by a system of ordinary and partial differential equations:

(1) $$\left\{\begin{array}{ll} & \frac{dS\left(t\right)}{dt}=p_{1}\mu A-\mu S\left(t\right)-\beta \left(M\left(t\right)\right)S\left(t\right)I\left(t\right)+\int_{0}^{\infty }w\left(a\right)v\left(t,a\right)\;da,\\ & \frac{\partial v\left(t,a\right)}{\partial t}+\frac{\partial v\left(t,a\right)}{\partial a}=-\left(\sigma \left(a\right)\beta \left(M\left(t\right)\right)I\left(t\right)+w\left(a\right)+\mu \right)v\left(t,a\right),\\ & \frac{\partial e\left(t,b\right)}{\partial t}+\frac{\partial e\left(t,b\right)}{\partial b}=-\left(\delta \left(b\right)+\mu \right)e\left(t,b\right),\\ & \frac{dI\left(t\right)}{dt}=\int_{0}^{\infty }\delta \left(b\right)e\left(t,b\right)\;db-\left(\mu +\gamma +v\right)I\left(t\right),\\ & \frac{dR\left(t\right)}{dt}=\gamma I\left(t\right)-\mu R\left(t\right),\\ & \frac{dM\left(t\right)}{dt}=u_{1}\alpha I\left(t\right)-dM\left(t\right). \end{array}\right)$$

Boundary conditions:

(2) $$v\left(t,0\right)=p_{2}\mu A,\;\;\;\;\;\;e\left(t,0\right)=\beta \left(M\left(t\right)\right)S\left(t\right)I\left(t\right)+\int_{0}^{\infty }\sigma \left(a\right)M\left(t\right)v\left(t,a\right)\;da.$$

Initial conditions:

(3) $$\begin{array}{c} S\left(0\right)=S_{0},V\left(0,a\right)=v_{0}\left(a\right),E\left(0,b\right)=e_{0}\left(b\right),I\left(0\right)=I_{0},R\left(0\right)=R_{0},M\left(0\right)=M_{0}. \end{array}$$

Here, $S_{0},I_{0},R_{0},M_{0}\in R^{+}$, $v_{0}\left(a\right),e_{0}\left(a\right)\in L_{1}^{+}\left(0,\infty \right)$. Here, $L_{1}^{+}\left(0,\infty \right)$ is the space of non-negative and Lebesgue integrable functions on (0, *∞*).

To have biological significance and to mathematically handle system (1), we make the following assumptions on the key functions *σ*(*a*), *w*(*a*), and *δ*(*b*).

Assumption 2.1 Consider system (1), and assume:

(i) $\sigma \left(a\right),w\left(a\right),\delta \left(b\right)\in L_{1}^{+}\left(0,\infty \right)$, with their respective essential upper bounds being $\bar{\sigma },\bar{w},\bar{\delta }$, that is,

$$\bar{\sigma }=\underset{a\in \left[0,\infty \right)}{\text{ess.sup}}\sigma \left(a\right)<\infty ,\;\bar{w}=\underset{a\in \left[0,\infty \right)}{\text{ess.sup}}w\left(a\right)<\infty ,\;\bar{\delta }=\underset{b\in \left[0,\infty \right)}{\text{ess.sup}}\delta \left(b\right)<\infty .$$

(ii) *σ*(*a*), *w*(*a*), and *δ*(*b*) are Lipschitz continuous, with Lipschitz constants *M*_*σ*_, *M*_*w*_, and *M*_*δ*_, respectively.
(iii) For all *a*, *b* ≥ 0, there exists *μ*_0_ ∈ (0, *μ*], such that *w*(*a*), *δ*(*b*) ≥ *μ*_0_.
(iv) *a* ∈ [0, *a*^+^] and *b* ∈ [0, *b*^+^], where *a*^+^ and *b* ^+^ are the maximum vaccination age and the maximum possible latent period, respectively. If *a*^+^, *b*^+^ = *∞*, then for all sufficiently large *a*, *b*, we have *v*(*t*, *a*) = 0 and *e*(*t*, *b*) = 0 for sufficiently large a,b captures finite-support (or fast-decaying) age distributions observed in practice and ensures the well-posedness of the PDE terms.
(v) 0 ≤ *u*_1_, *d* < 1.
(vi) *β*(0) = *β*_0_ > 0, and for *M* > 0, we have *β*(*M*) > 0. Define the space of functions *X* as

$$X=R^{+}\times L_{1}^{+}\left(0,\infty \right)\times L_{1}^{+}\left(0,\infty \right)\times R^{+}\times R^{+}\times R^{+}.$$

The norm on *X* is given by

$${\left\|\left(x_{1},x_{2},x_{3},x_{4},x_{5},x_{6}\right)\right\|}_{X}=\left|x_{1}\right|+\int_{0}^{\infty }\left|x_{2}\left(a\right)\right|\;da+\int_{0}^{\infty }\left|x_{3}\left(b\right)\right|\;db+\left|x_{4}\right|+\left|x_{5}\right|+\left|x_{6}\right|.$$

The initial conditions (3), which belong to the positive cone of *X*, can be rewritten as

$$\chi_{0}=\left(S_{0},v_{0}\left(\cdot \right),e_{0}\left(\cdot \right),I_{0},R_{0},M_{0}\right)\in X.$$

By the standard theory of functional differential equations, it can be verified that system (1) with the initial conditions (3) has a unique non-negative solution.

Thus, we can obtain a continuous semiflow associated with system (1), that is, the semiflow generated by system (1) is $\Phi :R^{+}\times X\to X$, given by

$$\Phi \left(t,x_{0}\right)=\Phi_{t}\left(x_{0}\right)=\left(S\left(t\right),v\left(t,\cdot \right),e\left(t,\cdot \right),I\left(t\right),R\left(t\right),M\left(t\right)\right),\;t\geq 0,\;x_{0}\in X,$$

and

(4) $$\begin{array}{ll} {\left\|\Phi_{t}\left(x_{0}\right)\right\|}_{X} & ={\left\|\left(S\left(t\right),v\left(t,\cdot \right),e\left(t,\cdot \right),I\left(t\right),R\left(t\right),M\left(t\right)\right)\right\|}_{X}\\ & =\left|S\left(t\right)\right|+\int_{0}^{\infty }\left|v\left(t,a\right)\right|\;da+\int_{0}^{\infty }\left|e\left(t,b\right)\right|\;db+\left|I\left(t\right)\right|+\left|R\left(t\right)\right|+\left|M\left(t\right)\right|. \end{array}$$

For convenience in notation, for *a*, *b* ≥ 0, we denote

$$\begin{array}{llllll} & k=\mu +\gamma +v, & & & \\ & \epsilon \left(s\right)=\sigma \left(s\right)\beta \left(M\right)+w\left(s\right)+\mu , & \rho_{1}\left(a\right) & =\exp\left(-\int_{0}^{a}\epsilon \left(s\right)\;ds\right), & \theta_{1} & =\int_{0}^{\infty }\sigma \left(a\right)\rho_{1}\left(a\right)\;da,\\ & \epsilon^{*}\left(a\right)=w\left(a\right)+\mu , & \rho_{1}^{*}\left(a\right) & =\exp\left(\int_{0}^{a}\epsilon^{*}\left(s\right)\;ds\right), & \\ & \eta \left(b\right)=\delta \left(b\right)+\mu , & \rho_{2}\left(b\right) & =\exp\left(-\int_{0}^{b}\eta \left(s\right)\;ds\right), & \theta_{2} & =\int_{0}^{\infty }\delta \left(b\right)\rho_{2}\left(b\right)\;db. \end{array}$$

By applying the Volterra formula and solving the partial differential equations in system (1) along the characteristic lines *t* − *a* = const and *t* − *b* = const, we can obtain

(5) $$\begin{array}{c} v\left(t,a\right)=\left\{\begin{array}{l} v\left(t-a,0\right)exp\left(-\int_{0}^{t}\epsilon \left(s\right)ds\right)=v\left(t-a,0\right)\rho_{1}\left(a\right),\;\;\;\;\;\;\;\;\;t\geq a.\;\\ v_{0}\left(a-t\right)exp\left(-\int_{a-t}^{a}\epsilon \left(s\right)ds\right)=v_{0}\left(a-t\right)\frac{\rho_{1}\left(a\right)}{\rho_{1}\left(a-t\right)},\;\;\;\;\;\;\;\;\;\;t<a.\; \end{array}\right) \end{array}$$

and

(6) $$\begin{array}{c} e\left(t,b\right)=\left\{\begin{array}{l} e\left(t-b,0\right)exp\left(-\int_{0}^{t}\eta \left(s\right)ds\right)=e\left(t-b,0\right)\rho_{2}\left(b\right),\;\;\;\;\;\;\;\;\;\;t\geq b.\;\\ e_{0}\left(b-t\right)exp\left(-\int_{b-t}^{b}\eta \left(s\right)ds\right)=e_{0}\left(b-t\right)\frac{\rho_{2}\left(b\right)}{\rho_{2}\left(b-t\right)},\;\;\;\;\;\;\;\;\;\;t<b.\; \end{array}\right) \end{array}$$

here,

$$v\left(t-a,0\right)=p_{2}\mu A,\;e\left(t-b,0\right)=\beta \left(M\left(t-b\right)\right)S\left(t-b\right)I\left(t-b\right)+\int_{0}^{\infty }\sigma \left(a\right)M\left(t-b\right)v\left(t-b,a\right)\;da.$$

Thus, system (1) can be rewritten as

(7) $$\left\{\begin{array}{l} \frac{dS\left(t\right)}{dt}=p_{1}\mu A-\mu S\left(t\right)-\beta \left(M\left(t\right)\right)S\left(t\right)I\left(t\right)+\int_{0}^{\infty }w\left(a\right)v\left(t,a\right)da.\;\\ v\left(t,a\right)=\left\{\begin{array}{l} v\left(t-a,0\right)exp\left(-\int_{0}^{t}\epsilon \left(s\right)ds\right)=v\left(t-a,0\right)\rho_{1}\left(a\right),t\geq a.\;\\ v_{0}\left(a-t\right)exp\left(-\int_{a-t}^{a}\epsilon \left(s\right)ds\right)=v_{0}\left(a-t\right)\frac{\rho_{1}\left(a\right)}{\rho_{1}\left(a-t\right)},t<a.\; \end{array}\right)\;\\ e\left(t,b\right)=\left\{\begin{array}{l} e\left(t-b,0\right)exp\left(-\int_{0}^{t}\eta \left(s\right)ds\right)=e\left(t-b,0\right)\rho_{2}\left(b\right),t\geq b.\;\\ e_{0}\left(b-t\right)exp\left(-\int_{b-t}^{b}\eta \left(s\right)ds\right)=e_{0}\left(b-t\right)\frac{\rho_{2}\left(b\right)}{\rho_{2}\left(b-t\right)},t<b.\; \end{array}\right)\;\\ \frac{dI\left(t\right)}{dt}=\int_{0}^{\infty }\delta \left(b\right)e\left(t,b\right)db-kI\left(t\right).\;\\ \frac{dR\left(t\right)}{dt}=\gamma I\left(t\right)-\mu R\left(t\right).\;\\ \frac{dM\left(t\right)}{dt}=u_{1}\alpha I\left(t\right)-dM\left(t\right).\; \end{array}\right)$$

Finally, the state space of system (1) is defined as

$$\Omega =\left\{\begin{array}{c} \left(S\left(t\right),v\left(t,\cdot \right),e\left(t,\cdot \right),I\left(t\right),R\left(t\right),M\left(t\right)\right)\in X:\\ S\left(t\right)+\int_{0}^{\infty }v\left(t,a\right)da+\int_{0}^{\infty }e\left(t,b\right)db+I\left(t\right)+R\left(t\right)\leq A,0<M\left(t\right)<\int_{0}^{\infty }I\left(t\right)dt \end{array}\right\}.$$

The following proposition can be used to prove that it is positively invariant.

Proposition 2.1 *Consider the system* (1), *then*

(i) Ω *is positively invariant for* Φ, *that is*, *for all* *t* ≥ 0 *and* *x*_0_ ∈ Ω, *we have* Φ(*t*, *x*_0_) ∈ Ω.
(ii) Φ *is point dissipative*, *and* Ω *attracts all points in* *X*. *Proof*: *For* (4), *we have*

(8) $$\frac{d}{dt}{\left\|\Phi_{t}\left(x_{0}\right)\right\|}_{X}=\frac{dS}{dt}+\frac{d}{dt}\int_{0}^{\infty }v\left(t,a\right)\;da+\frac{d}{dt}\int_{0}^{\infty }e\left(t,b\right)\;db+\frac{dI}{dt}+\frac{dR}{dt}+\frac{dM}{dt}.$$

From (5), we know that

$$\begin{array}{ll} \frac{d}{dt}\int_{0}^{\infty }v\left(t,a\right)\;da & =\frac{d}{dt}\int_{0}^{t}p_{2}\mu A\rho_{1}\left(a\right)\;da+\frac{d}{dt}\int_{t}^{\infty }v_{0}\left(a-t\right)\frac{\rho_{1}\left(a\right)}{\rho_{1}\left(a-t\right)}\;da\\ & =\frac{d}{dt}\int_{0}^{t}p_{2}\mu A\rho_{1}\left(t-u\right)\;du+\frac{d}{dt}\int_{0}^{\infty }v_{0}\left(u\right)\frac{\rho_{1}\left(t+u\right)}{\rho_{1}\left(u\right)}\;du\\ & =p_{2}\mu A\rho_{1}\left(0\right)+\int_{0}^{t}p_{2}\mu A\frac{d}{dt}\rho_{1}\left(t-u\right)\;du+\int_{0}^{\infty }v_{0}\left(u\right)\frac{\frac{d}{dt}\rho_{1}\left(t+u\right)}{\rho_{1}\left(u\right)}\;du. \end{array}$$

Noting that *ρ*_1_(0) = 1 and $\frac{d}{da}\rho_{1}\left(a\right)=-\epsilon \left(a\right)\rho_{1}\left(a\right)$, we get

(9) $$\frac{d}{dt}\int_{0}^{\infty }v\left(t,a\right)\;da=p_{2}\mu A-\int_{0}^{\infty }\epsilon \left(a\right)v\left(t,a\right)\;da.$$

Similarly, we get

(10) $$\frac{d}{dt}\int_{0}^{\infty }e\left(t,b\right)\;db=\beta \left(M\left(t\right)\right)S\left(t\right)I\left(t\right)+\int_{0}^{\infty }\sigma \left(a\right)\beta \left(M\left(t\right)\right)v\left(t,a\right)\;da-\int_{0}^{\infty }\eta \left(b\right)e\left(t,b\right)\;db.$$

Adding (9), (10), and the first, fourth, and fifth equations of system (1), we get

$$\begin{array}{ll} & \;\frac{dS}{dt}+\frac{d}{dt}\int_{0}^{\infty }v\left(t,a\right)\;da+\frac{d}{dt}\int_{0}^{\infty }e\left(t,b\right)\;db+\frac{dI}{dt}+\frac{dR}{dt}\\ & =\mu \left(A-\left(S\left(t\right)+\int_{0}^{\infty }v\left(t,a\right)\;da+\int_{0}^{\infty }e\left(t,b\right)\;db+I\left(t\right)+R\left(t\right)\right)\right). \end{array}$$

Therefore, $\underset{t\to \infty }{\lim}S\left(t\right)+\int_{0}^{\infty }v\left(t,a\right)\;da+\int_{0}^{\infty }e\left(t,b\right)\;db+I\left(t\right)+R\left(t\right)\leq A$. Furthermore, from the sixth equation of system (1), we have $\frac{dM}{dt}=u_{1}\alpha I\left(t\right)-dM\left(t\right)\leq A$ which implies that $\underset{t\to \infty }{\mathrm{limsup}}{\left\|\Phi_{t}\left(x_{0}\right)\right\|}_{X}\leq 2A$. Thus, for any *x*_0_ ∈ Ω, and for all *t* ≥ 0, any solution of system (1) satisfies Φ_*t*_(*x*_0_) ∈ Ω. It is easy to verify the positive invariance of Ω under the solution semiflow Φ, and that Ω attracts every point in *X*, i.e., (ii) holds.

By applying Assumption 2.1 and Proposition 2.1, the following proposition is established.

Proposition 2.2 *For constants* *C*_1_ ≥ *A* *and* *C*_2_ ≥ *a*^+^*I*(*t*), *if* *x*_0_ ∈ *X* *and* ‖*x*_0_‖_*X*_ ≤ *C*_1_, *then the following statements hold for* *t* ≥ 0:

(i) $0\leq S\left(t\right),\int_{0}^{\infty }v\left(t,a\right)\;da,\int_{0}^{\infty }e\left(t,b\right)\;db,I\left(t\right),R\left(t\right)\leq C_{1}$.
(ii) $v\left(t,0\right)\leq \beta C_{1}^{2}$, *e*(*t*, 0) ≤ *ξC*_1_.

## 3 Existence of positive equilibrium

Consider the steady state of system (1). The steady state $\left(S^{*},v^{*}\left(\cdot \right),e^{*}\left(\cdot \right),I^{*},R^{*},M^{*}\right)$ of system (1) satisfies the following equations:

(11) $$\left\{\begin{array}{ll} & 0=p_{1}\mu A-\mu S^{*}-\beta \left(M^{*}\right)S^{*}I^{*}+\int_{0}^{\infty }w\left(a\right)v^{*}\left(a\right)\;da,\\ & \frac{dv^{*}\left(a\right)}{da}=-\epsilon \left(a\right)v^{*}\left(a\right),\\ & \frac{de^{*}\left(b\right)}{db}=-\eta \left(b\right)e^{*}\left(b\right),\\ & 0=\int_{0}^{\infty }\delta \left(b\right)e^{*}\left(b\right)\;db-kI^{*},\\ & 0=\gamma I^{*}-\mu R^{*},\\ & 0=u_{1}\alpha I^{*}-dM^{*}. \end{array}\right)$$

From the second equation of (11), we get

$$\int_{0}^{a}\frac{dv^{*}\left(\tau \right)}{v^{*}\left(\tau \right)}=\int_{0}^{a}-\epsilon \left(s\right)\;ds,$$

and with the boundary condition *v*∗(0) = *p*_2_*μA*, we obtain

(12) $$v^{*}\left(a\right)=p_{2}\mu A\exp\left(-\int_{0}^{a}\epsilon \left(s\right)\;ds\right)=p_{2}\mu A\rho_{1}\left(a\right).$$

Similarly, from the third equation of (11) and the boundary condition $E^{*}\left(0\right)=\beta \left(M^{*}\right)S^{*}I^{*}+\int_{0}^{\infty }\sigma \left(a\right)\beta \left(M^{*}\right)v^{*}\left(a\right)\;da$, we get

(13) $$\begin{array}{ll} e^{*}\left(b\right) & =\left(\beta \left(M^{*}\right)S^{*}I^{*}+\int_{0}^{\infty }\sigma \left(a\right)\beta \left(M^{*}\right)v^{*}\left(a\right)\;da\right)\exp\left(-\int_{0}^{b}\eta \left(s\right)\;ds\right)\\ & =\left(\beta \left(M^{*}\right)S^{*}I^{*}+\int_{0}^{\infty }\sigma \left(a\right)\beta \left(M^{*}\right)v^{*}\left(a\right)\;da\right)\rho_{2}\left(b\right). \end{array}$$

Substituting (12) into the first equation of (11), we get

(14) $$S^{*}=\frac{p_{1}A+\int_{0}^{\infty }w\left(a\right)v^{*}\left(a\right)\;da}{\mu +\beta \left(M^{*}\right)}.$$

If *I*∗ = 0, then from the fourth, fifth, and sixth equations of (11), we have *e*∗(*b*) = 0, *R*∗ = 0, *M*∗ = 0. Further, combining (12) and (14), we get

(15) $$S^{0}=A\left(p_{1}+p_{2}\int_{0}^{\infty }w\left(a\right)\rho_{1}\left(a\right)\;da\right),\;v^{0}\left(a\right)=p_{2}\mu A\rho_{1}\left(a\right).$$

Therefore, system (1) always has a disease-free equilibrium $E^{0}=\left(S^{0},v^{0}\left(a\right),0,0,0,0\right)$.

To determine the existence of the endemic equilibrium of system (1), we first define the basic reproduction number based on biological significance as

$$R_{0}=\frac{\beta_{0}S^{0}}{\mu +\gamma +\nu }\left(\int_{0}^{\infty }\delta \left(b\right)e^{-\mu b-\int_{0}^{b}\delta \left(s\right)\;ds}\;db\right).$$

From an epidemiological perspective, the vaccination reproduction number *R*_0_ represents the expected number of new infection cases produced by a typical infected individual during the entire infection period $\frac{1}{\mu +\gamma +\nu }$ in a population where a small fraction of susceptible individuals are vaccinated (Anderson & May 1990; Hethcote, 2000).

Thus, *R*_0_ serves as a threshold parameter for the stability of the equilibrium points of system (1).

If *I*∗ ≠ 0, then from the first equation of (11), we have

$$0=p_{1}\mu A-\mu S^{*}-\beta \left(M^{*}\right)S^{*}I^{*}+\int_{0}^{\infty }w\left(a\right)v^{*}\left(a\right)\;da.$$

This equation describes the change in the number of susceptible individuals *S*∗. To simplify, we first calculate the integral term using the expression for *v*∗(*a*), $v^{*}\left(a\right)=p_{2}\mu A\exp\left(-\int_{0}^{a}\epsilon \left(s\right)\;ds\right)$, to get

$$\int_{0}^{\infty }w\left(a\right)v^{*}\left(a\right)\;da=\int_{0}^{\infty }w\left(a\right)p_{2}\mu A\exp\left(-\int_{0}^{a}\epsilon \left(s\right)\;ds\right)\;da.$$

Substituting this into the equation above, we get the steady-state solution for the susceptible individuals

$$S^{*}=\frac{p_{1}\mu A+\int_{0}^{\infty }w\left(a\right)v^{*}\left(a\right)\;da}{\mu +\beta \left(M^{*}\right)I^{*}}.$$

From the second equation of (11), $\frac{dv^{*}\left(a\right)}{da}=-\epsilon \left(a\right)v^{*}\left(a\right)$, and the boundary condition *v*∗(0) = *p*_2_*μA*, we get

$$v^{*}\left(a\right)=p_{2}\mu A\exp\left(-\int_{0}^{a}\epsilon \left(s\right)\;ds\right).$$

From the third equation of (11), $\frac{de^{*}\left(b\right)}{db}=-\eta \left(b\right)e^{*}\left(b\right)$, and the initial condition.

$e^{*}\left(0\right)=\beta \left(M^{*}\right)S^{*}I^{*}+\int_{0}^{\infty }\sigma \left(a\right)\beta \left(M^{*}\right)v^{*}\left(a\right)\;da$, we get

$$e^{*}\left(b\right)=\left(\beta \left(M^{*}\right)S^{*}I^{*}+\int_{0}^{\infty }\sigma \left(a\right)\beta \left(M^{*}\right)v^{*}\left(a\right)\;da\right)\exp\left(-\int_{0}^{b}\eta \left(s\right)\;ds\right).$$

From the fourth equation of (11), we get the expression for *I*∗. First, substituting *e*∗(*b*) into it, we get

$$\int_{0}^{\infty }\delta \left(b\right)e^{*}\left(b\right)\;db=\int_{0}^{\infty }\delta \left(b\right)\left(\beta \left(M^{*}\right)S^{*}I^{*}+\int_{0}^{\infty }\sigma \left(a\right)\beta \left(M^{*}\right)v^{*}\left(a\right)\;da\right)\exp\left(-\int_{0}^{b}\eta \left(s\right)\;ds\right)\;db.$$

Thus, we get the infected individuals

$$I^{*}=\frac{1}{k}\int_{0}^{\infty }\delta \left(b\right)e^{*}\left(b\right)\;db.$$

From the fifth equation of (11), we get $R^{*}=\frac{\gamma I^{*}}{\mu }$. From the sixth equation of (11), we get 0 = *u*_1_*αI*∗ − *dM*∗. Thus, $M^{*}=\frac{u_{1}\alpha I^{*}}{d}$.

Therefore, model (1) has a unique endemic equilibrium *E*∗ = (*S*∗, *v*∗(*a*, *t*), *e*∗(*a*, *t*), *I*∗, *R*∗, *M*∗).

## 4 Main results

This section discusses the global stability of the equilibrium points and the uniform persistence of system (1). Before the discussion, some preparatory work is done.

To analyze the global stability of system (1), we introduce the Volterra-type function

g(x)=x−1−lnx,x>0.

It is clear that when *x* > 0, *g*(*x*) ≥ 0, and $g^{\prime }\left(x\right)=1-\frac{1}{x}$, so *g*(*x*) has a global minimum at *x* = 1 with *g*(1) = 0.

### 4.1 Local stability of steady-state solutions

Theorem 4.1 *When* *R*_0_ < 1, *the disease-free equilibrium* *E*_0_ *is locally asymptotically stable*; *when* *R*_0_ > 1, *the disease-free equilibrium* *E*_0_ *is unstable*.

*Proof*: *First*, *introduce the following variables* $x_{1}\left(t\right)=S\left(t\right)-S^{0},x_{2}\left(t,a\right)=v\left(t,a\right)-v^{0}\left(a\right),x_{3}\left(t,b\right)=e\left(t,b\right),x_{4}\left(t\right)=I\left(t\right),x_{5}\left(t\right)=R\left(t\right),x_{6}\left(t\right)=M\left(t\right)$.

*Linearizing system* (1) *around the disease-free equilibrium* *E*_0_ *yields the following system*:

(16) $$\left\{\begin{array}{ll} & \frac{dx_{1}\left(t\right)}{dt}=-\mu x_{1}\left(t\right)-x_{6}\left(t\right)x_{1}\left(t\right)+\int_{0}^{\infty }w\left(a\right)x_{2}\left(t,a\right)\;da,\\ & \frac{\partial x_{2}\left(t,a\right)}{\partial t}+\frac{\partial x_{2}\left(t,a\right)}{\partial a}=-\left(\sigma \left(a\right)x_{6}\left(t\right)+w\left(a\right)+\mu \right)x_{2}\left(t,a\right),\\ & \frac{\partial x_{3}\left(t,b\right)}{\partial t}+\frac{\partial x_{3}\left(t,b\right)}{\partial b}=-\left(\delta \left(b\right)+\mu \right)x_{3}\left(t,b\right),\\ & \frac{dx_{4}\left(t\right)}{dt}=\int_{0}^{\infty }\delta \left(b\right)x_{3}\left(t,b\right)\;db-\left(\mu +\gamma +v\right)x_{4}\left(t\right),\\ & \frac{dx_{5}\left(t\right)}{dt}=\gamma x_{4}\left(t\right)-\mu x_{5}\left(t\right),\\ & \frac{dx_{6}\left(t\right)}{dt}=u_{1}\int_{0}^{\infty }\alpha \left(a\right)x_{4}\left(t\right)\;da-dx_{6}\left(t\right),\\ & x_{2}\left(t,0\right)=0,\\ & x_{3}\left(t,0\right)=\beta_{0}S_{0}I_{0}+\int_{0}^{\infty }\sigma \left(a\right)x_{6}\left(t\right)x_{2}\left(a\right)\;da. \end{array}\right)$$

*Assume* $x_{1}\left(t\right)=x_{1}^{0}e^{\lambda t},x_{2}\left(t,a\right)=x_{2}^{0}\left(a\right)e^{\lambda t},x_{3}\left(t,b\right)=x_{3}^{0}\left(b\right)e^{\lambda t},x_{4}\left(t\right)=x_{4}^{0}e^{\lambda t},x_{5}\left(t\right)=x_{5}^{0}e^{\lambda t},x_{6}\left(t\right)=x_{6}^{0}e^{\lambda t}$.

*Substituting these into* (16), *we get*

(17) $$\lambda x_{1}^{0}=-\mu x_{1}^{0}-x_{6}^{0}x_{1}^{0}+\int_{0}^{\infty }w\left(a\right)x_{2}^{0}\left(a\right)\;da.$$

(18) $$\left\{\begin{array}{ll} & \lambda x_{2}^{0}\left(a\right)+\frac{dx_{2}^{0}\left(a\right)}{da}=-\left(\sigma \left(a\right)x_{6}^{0}+w\left(a\right)+\mu \right)x_{2}^{0}\left(a\right),\\ & x_{2}^{0}\left(0\right)=0. \end{array}\right)$$

(19) $$\left\{\begin{array}{ll} & \lambda x_{3}^{0}\left(b\right)+\frac{dx_{3}^{0}\left(b\right)}{db}=-\left(\delta \left(b\right)+\mu \right)x_{3}^{0}\left(b\right),\\ & x_{3}^{0}\left(0\right)=\beta_{0}S_{0}I_{0}+\int_{0}^{\infty }\sigma \left(a\right)x_{6}^{0}x_{2}^{0}\left(a\right)\;da. \end{array}\right)$$

(20) $$\lambda x_{4}^{0}=\int_{0}^{\infty }\delta \left(b\right)x_{3}^{0}\left(b\right)\;db-\left(\mu +\gamma +v\right)x_{4}^{0}.$$

(21) $$\lambda x_{5}^{0}=\gamma x_{4}^{0}-\mu x_{5}^{0}.$$

(22) $$\lambda x_{6}^{0}=u_{1}\int_{0}^{\infty }\alpha \left(a\right)x_{4}^{0}\;da-dx_{6}^{0}.$$

*Integrating the first equation of* (19) *from* 0 *to* *b*, *we get*

(23) $$x_{3}^{0}\left(b\right)=x_{3}^{0}\left(0\right)e^{-\left(\lambda +\mu \right)b-\int_{0}^{b}\delta \left(s\right)\;ds}=\left(\beta_{0}S_{0}I_{0}+\int_{0}^{\infty }\sigma \left(a\right)x_{6}^{0}x_{2}^{0}\left(a\right)\;da\right)e^{-\left(\lambda +\mu \right)b-\int_{0}^{b}\delta \left(s\right)\;ds}.$$

*Substituting* (23) *into* (20), *we get*

(24) $$\lambda x_{4}^{0}+\left(\mu +\gamma +v\right)x_{4}^{0}=x_{3}^{0}\left(0\right)\int_{0}^{\infty }\delta \left(b\right)e^{-\left(\lambda +\mu \right)b-\int_{0}^{b}\delta \left(s\right)\;ds}\;db.$$

*From the boundary condition* *x*_3_(*t*, 0) = *β*_0_*S*_0_*x*_4_(*t*), *we get*

(25) $$x_{3}^{0}\left(0\right)=\beta_{0}S_{0}x_{4}^{0}.$$

*Substituting* (25) *into* (24), *we get*

(26) $$\lambda x_{4}^{0}+kx_{4}^{0}=\beta_{0}S_{0}x_{4}^{0}\int_{0}^{\infty }\delta \left(b\right)e^{-\left(\lambda +\mu \right)b-\int_{0}^{b}\delta \left(s\right)\;ds}\;db,\;\;k=\mu +\gamma +v.$$

*Dividing by* $x_{4}^{0}\neq 0$, *we get the characteristic equation*

(27) $$\lambda +k=\beta_{0}S_{0}\int_{0}^{\infty }\delta \left(b\right)e^{-\left(\lambda +\mu \right)b-\int_{0}^{b}\delta \left(s\right)\;ds}\;db.$$

*We examine whether all* *λ* *have negative real parts*. *Setting* *λ* = 0 *in* (27), *we get*

(28) $$k=\beta_{0}S_{0}\int_{0}^{\infty }\delta \left(b\right)e^{-\mu b-\int_{0}^{b}\delta \left(s\right)\;ds}\;db.$$

*Define*

$$\theta_{2}=\int_{0}^{\infty }\delta \left(b\right)e^{-\mu b-\int_{0}^{b}\delta \left(s\right)\;ds}\;db\Rightarrow \frac{\beta_{0}S_{0}\theta_{2}}{k}=R_{0}.$$

*Therefore*, *if* *R*_0_ < 1, *then the right-hand side* (*RHS*) *is less than* *k*, *implying that when* *λ* = 0, *the right-hand side of* (28) *is less than the left-hand side*, *which suggests the existence of a solution with* *λ* < 0. *If* *R*_0_ > 1, *then the RHS is greater than* *k*, *indicating the existence of* *λ* *with positive real parts*, *rendering the system unstable*.

*In summary*, *when* *R*_0_ < 1, *the disease-free equilibrium* *E*_0_ *is locally asymptotically stable*. *When* *R*_0_ > 1, *the disease-free equilibrium* *E*_0_ *is unstable*.

Theorem 4.2 *When* *R*_0_ < 1, *the disease-free equilibrium* *E*_0_ *is globally asymptotically stable*.

*Proof*: *Consider the following Lyapunov function*, *defined as* *V*_0_ = *L*_*s*_ + *L*_*v*_ + *L*_*e*_ + *L*_*i*_, *where*

$$\begin{array}{llll} L_{s}\left(t\right) & =\rho_{1}S^{0}g\left(\frac{S}{S^{0}}\right), & L_{v}\left(t\right) & =\rho_{1}\int_{0}^{\infty }v^{0}\left(a\right)g\left(\frac{v\left(t,a\right)}{v^{0}\left(a\right)}\right)da,\\ L_{e}\left(t\right) & =\int_{0}^{\infty }v_{2}\left(b\right)e\left(t,b\right)db, & L_{i}\left(t\right) & =I\left(t\right). \end{array}$$

*Combining the solutions of system* (1), *the derivative of* *L*_*s*_ *is*

(29) $$\begin{array}{ll} \frac{dL_{s}\left(t\right)}{dt} & =\rho_{1}S^{0}\left(1-\frac{S^{0}}{S}\right)\frac{1}{S^{0}}\left[p_{1}\mu A\left(1-\frac{S^{0}}{S}\right)-\beta \left(M\right)SI+\int_{0}^{\infty }w\left(a\right)v^{0}\left(a\right)\left(\frac{v\left(t,a\right)}{v^{0}\left(a\right)}-\frac{S}{S^{0}}\right)da\right]\\ & =\rho_{1}p_{1}\mu A\left(2-\frac{S}{S^{0}}-\frac{S^{0}}{S}\right)+\rho_{1}\int_{0}^{\infty }w\left(a\right)v^{0}\left(a\right)\left(\frac{v\left(t,a\right)}{v^{0}\left(a\right)}-\frac{S}{S^{0}}-\frac{S^{0}v\left(t,a\right)}{Sv^{0}\left(a\right)}+1\right)da+L_{s}^{+}. \end{array}$$

Here, $L_{s}^{+}=-\rho_{1}\beta \left(M\right)SI+\rho_{1}\beta \left(M\right)S^{0}I^{0}$.

*Now*, *we start to calculate the derivative of* *L*_*v*_(*t*) *along the solutions of system* (1),

$$\begin{array}{ll} \frac{dL_{v}\left(t\right)}{dt} & =\rho_{1}\int_{0}^{\infty }v^{0}\left(a\right)\frac{\partial }{\partial t}g\left(\frac{v\left(t,a\right)}{v^{0}\left(a\right)}\right)da\\ & =-\rho_{1}\int_{0}^{\infty }v^{0}\left(a\right)\left(1-\frac{v^{0}\left(a\right)}{v\left(t,a\right)}\right)\frac{1}{v^{0}\left(a\right)}\left[\frac{\partial }{\partial a}v\left(t,a\right)+\left(\sigma \left(a\right)\beta \left(M\right)+w\left(a\right)+\mu \right)v\left(t,a\right)\right]da\\ & =-\rho_{1}\int_{0}^{\infty }v^{0}\left(a\right)\left(\frac{v\left(t,a\right)}{v^{0}\left(a\right)}-1\right)\left[\frac{v_{a}\left(t,a\right)}{v\left(t,a\right)}+\left(\sigma \left(a\right)\beta \left(M\right)+w\left(a\right)+\mu \right)\right]da\\ & =-\rho_{1}\int_{0}^{\infty }v^{0}\left(a\right)\left(\frac{v\left(t,a\right)}{v^{0}\left(a\right)}-1\right)\left(\frac{v_{a}\left(t,a\right)}{v\left(t,a\right)}+w\left(a\right)+\mu \right)da+L_{v}^{+}. \end{array}$$

Here, *v*_*a*_(*t*, *a*) *denotes* $\frac{\partial }{\partial a}v\left(t,a\right)$, *and*

$$L_{v}^{+}=-\rho_{1}\int_{0}^{\infty }\sigma \left(a\right)\beta \left(M\right)v\left(t,a\right)da+\rho_{1}\int_{0}^{\infty }\sigma \left(a\right)\beta \left(M\right)v^{0}\left(a\right)da.$$

*From* $\frac{dv^{0}\left(a\right)}{da}=-\left(w\left(a\right)+\mu \right)v^{0}\left(a\right)$, *we get*

$$\frac{\partial }{\partial a}g\left(\frac{v\left(t,a\right)}{v^{0}\left(a\right)}\right)=\left(\frac{v\left(t,a\right)}{v^{0}\left(a\right)}-1\right)\left(\frac{v_{a}\left(t,a\right)}{v\left(t,a\right)}+w\left(a\right)+\mu \right).$$

*By integration by parts*, *combined with* $g\left(\frac{v\left(t,0\right)}{v^{0}\left(0\right)}\right)=g\left(1\right)=0$, *we can derive*

$$\begin{array}{ll} \int_{0}^{\infty }v^{0}\left(a\right)\frac{\partial }{\partial a}g\left(\frac{v\left(t,a\right)}{v^{0}\left(a\right)}\right)da & =v^{0}\left(a\right){\left(g\left(\frac{v\left(t,a\right)}{v^{0}\left(a\right)}\right)\right|}_{a=0}^{a=\infty }-\int_{0}^{\infty }g\left(\frac{v\left(t,a\right)}{v^{0}\left(a\right)}\right)\frac{dv^{0}\left(a\right)}{da}da\\ & =v^{0}\left(a\right){\left(g\left(\frac{v\left(t,a\right)}{v^{0}\left(a\right)}\right)\right|}_{a=\infty }+\int_{0}^{\infty }v^{0}\left(a\right)\left(w\left(a\right)+\mu \right)g\left(\frac{v\left(t,a\right)}{v^{0}\left(a\right)}\right)da. \end{array}$$

*Further*, *we have*

(30) $$\begin{array}{ll} \frac{dL_{v}\left(t\right)}{dt} & =-\rho_{1}\int_{0}^{\infty }v^{0}\left(a\right)\frac{\partial }{\partial a}g\left(\frac{v\left(t,a\right)}{v^{0}\left(a\right)}\right)da+L_{v}^{+}\\ & =-\rho_{1}v^{0}\left(a\right){\left(g\left(\frac{v\left(t,a\right)}{v^{0}\left(a\right)}\right)\right|}_{a=\infty }-\rho_{1}\int_{0}^{\infty }v^{0}\left(a\right)\left(w\left(a\right)+\mu \right)g\left(\frac{v\left(t,a\right)}{v^{0}\left(a\right)}\right)da+L_{v}^{+}. \end{array}$$

*The time derivative of* *L*_*e*_(*t*) *along the solutions of system* (1) *is*

$$\frac{dL_{e}\left(t\right)}{dt}=\int_{0}^{\infty }v_{2}\left(b\right)\frac{\partial }{\partial t}e\left(t,b\right)db=-\int_{0}^{\infty }v_{2}\left(b\right)\frac{\partial }{\partial b}e\left(t,b\right)db-\int_{0}^{\infty }v_{2}\left(b\right)\left(\delta \left(b\right)+\mu \right)e\left(t,b\right)db.$$

*By integration by parts*, *from system* (1), *we have*

$$\begin{array}{ll} \int_{0}^{\infty }v_{2}\left(b\right)\frac{\partial }{\partial b}e\left(t,b\right)db & ={\left(v_{2}\left(b\right)e\left(t,b\right)\right|}_{b=0}^{b=\infty }-\int_{0}^{\infty }e\left(t,b\right)\frac{dv_{2}\left(b\right)}{db}db\\ & ={\left(v_{2}\left(b\right)e\left(t,b\right)\right|}_{b=\infty }-\theta_{2}e\left(t,0\right)-\int_{0}^{\infty }e\left(t,b\right)\left[\left(\delta \left(b\right)+\mu \right)v_{2}\left(b\right)-\delta \left(b\right)\right]db. \end{array}$$

*Thus*, *we have*

(31) $$\frac{dL_{e}\left(t\right)}{dt}=-{\left(v_{2}\left(b\right)e\left(t,b\right)\right|}_{b=\infty }+\theta_{2}e\left(t,0\right)-\int_{0}^{\infty }\delta \left(b\right)e\left(t,b\right)db.$$

*Clearly*,

(32) $$\frac{dL_{i}\left(t\right)}{dt}=\frac{dI}{dt}=\int_{0}^{\infty }\delta \left(b\right)e\left(t,b\right)db-\left(\mu +\gamma +v\right)I\left(t\right).$$

*Based on the boundary conditions* (2), *from* (29) *to* (32), *we obtain*

$$\begin{array}{ll} \frac{dV_{0}\left(t\right)}{dt} & =\frac{dL_{s}\left(t\right)}{dt}+\frac{dL_{v}\left(t\right)}{dt}+\frac{dL_{e}\left(t\right)}{dt}+\frac{dL_{i}\left(t\right)}{dt}\\ & =\rho_{1}p_{1}\mu A\left(2-\frac{S}{S^{0}}-\frac{S^{0}}{S}\right)+\rho_{1}\int_{0}^{\infty }w\left(a\right)v^{0}\left(a\right)\left(\frac{v\left(t,a\right)}{v^{0}\left(a\right)}-\frac{S}{S^{0}}-\frac{S^{0}v\left(t,a\right)}{Sv^{0}\left(a\right)}+1\right)da\\ & \;-{\left(\rho_{1}v^{0}\left(a\right)g\left(\frac{v\left(t,a\right)}{v^{0}\left(a\right)}\right)\right|}_{a=\infty }-\rho_{1}\int_{0}^{\infty }v^{0}\left(a\right)\left(w\left(a\right)+\mu \right)g\left(\frac{v\left(t,a\right)}{v^{0}\left(a\right)}\right)da\\ & \;-{\left(v_{2}\left(b\right)e\left(t,b\right)\right|}_{b=\infty }+\theta_{2}\beta \left(M\right)S^{0}I^{0}+\rho_{1}\int_{0}^{\infty }\sigma \left(a\right)v^{0}\left(a\right)\beta \left(M\right)da-\left(\mu +\gamma +v\right)I\\ & =\rho_{1}p_{1}\mu A\left(2-\frac{S}{S^{0}}-\frac{S^{0}}{S}\right)-v^{0}\left(a\right){\left(g\left(\frac{v\left(t,a\right)}{v^{0}\left(a\right)}\right)\right|}_{a=\infty }-{\left(v_{2}\left(b\right)e\left(t,b\right)\right|}_{b=\infty }\\ & \;-\rho_{1}\mu \int_{0}^{\infty }v^{0}\left(a\right)g\left(\frac{v\left(t,a\right)}{v^{0}\left(a\right)}\right)da+L_{1}+L_{2}. \end{array}$$

*Here*,

$$\begin{array}{ll} L_{1} & =\rho_{1}\int_{0}^{\infty }w\left(a\right)v^{0}\left(a\right)\left(\frac{v\left(t,a\right)}{v^{0}\left(a\right)}-\frac{S}{S^{0}}-\frac{S^{0}v\left(t,a\right)}{Sv^{0}\left(a\right)}+1-\frac{v\left(t,a\right)}{v^{0}\left(a\right)}+1+\ln\frac{v\left(t,a\right)}{v^{0}\left(a\right)}\right)da\\ & =\rho_{1}\int_{0}^{\infty }w\left(a\right)v^{0}\left(a\right)\left(-\frac{S}{S^{0}}+1+\ln\frac{S}{S^{0}}-\frac{S^{0}v\left(t,a\right)}{Sv^{0}\left(a\right)}+1+\ln\frac{S^{0}v\left(t,a\right)}{Sv^{0}\left(a\right)}\right)da\\ & =-\rho_{1}\int_{0}^{\infty }w\left(a\right)v^{0}\left(a\right)\left(g\left(\frac{S}{S^{0}}\right)+g\left(\frac{S^{0}v\left(t,a\right)}{Sv^{0}\left(a\right)}\right)\right)da. \end{array}$$

$$\begin{array}{ll} L_{2} & =\theta_{2}\left(S^{0}+\int_{0}^{\infty }\sigma \left(a\right)v^{0}\left(a\right)\;da\right)\beta \left(M\right)I-\left(\mu +\gamma +v\right)I,\\ & =\left(\mu +\gamma +v\right)\left[\frac{\theta_{2}}{\mu +\gamma +v}\left(S^{0}+\int_{0}^{\infty }\sigma \left(a\right)v^{0}\left(a\right)\;da\right)\beta \left(M\right)-1\right]I. \end{array}$$

*Introducing a local reproduction number*,

$${\tilde{R}}_{0}\left(M\right)≔\frac{\beta \left(M\right)\left(S_{0}+\int_{0}^{\infty }\sigma \left(a\right)v_{0}\left(a\right)\;da\right)}{\mu +\gamma +\nu }.$$

*Then*,

$$L_{2}=\left(\mu +\gamma +\nu \right)\left({\tilde{R}}_{0}\left(M\right)-1\right)I.$$

*Therefore*, *we obtain*

$$\begin{array}{ll} \frac{dV_{0}\left(t\right)}{dt} & \leq \rho_{1}p_{1}\mu A\left(2-\frac{S}{S^{0}}-\frac{S^{0}}{S}\right)-\rho_{1}\mu \int_{0}^{\infty }v^{0}\left(a\right)g\left(\frac{v\left(t,a\right)}{v^{0}\left(a\right)}\right)da-v^{0}\left(a\right){\left(g\left(\frac{v\left(t,a\right)}{v^{0}\left(a\right)}\right)\right|}_{a=\infty }\\ & \;-{\left(v_{2}\left(b\right)e\left(t,b\right)\right|}_{b=\infty }-\rho_{1}\int_{0}^{\infty }w\left(a\right)v^{0}\left(a\right)\left(g\left(\frac{S}{S^{0}}\right)+g\left(\frac{S^{0}v\left(t,a\right)}{Sv^{0}\left(a\right)}\right)\right)da\\ & \;+\left(\mu +\gamma +v\right)\left({\tilde{R}}_{0}\left(M\right)-1\right)I. \end{array}$$

*Thus*, ${\tilde{R}}_{0}\left(M\right)<1$ *ensures that* $\frac{dV_{0}\left(t\right)}{dt}\leq 0$ *if and only if* *S* = *S*_0_, *v* = *v*_0_(*a*), *e* ≡ 0, *I* ≡ 0, *M* ≡ 0. *Furthermore*, *the equality holds if and only if* *S* = *S*^0^, *v*(*t*, *a*) = 0, *e*(*t*, *b*) = 0, *and* *I* = 0. *Therefore*, $M_{0}=\left\{E_{0}\right\}\subset \Omega$ *is the largest invariant subset where* $\frac{dV_{0}\left(t\right)}{dt}=0$. *According to the Lyapunov-Lasalle invariance principle* (Liu et al., 2007), *when* *R*_0_ < 1, *the disease-free equilibrium* *E*_0_ *is globally asymptotically stable*.

### 4.2 Uniform persistence

Next, we will use the following result about linear scalar Volterra integro-differential equations in the analysis.

Lemma 4.1 *Consider the following linear scalar Volterra integro-differential equation*:

(33) $$\frac{dy\left(t\right)}{dt}=\int_{0}^{\infty }h\left(\tau \right)y\left(t-\tau \right)\;d\tau -cy\left(t\right),\;y\left(0\right)>0.$$

Here, $h\left(\cdot \right)\in L_{+}^{1}\left(0,\infty \right)$, *c* > 0, *and* $\int_{0}^{\infty }h\left(\tau \right)\;d\tau >c$. *There exists a unique unbounded solution* *y*(*t*).

*To define the invariant set for uniform persistence*, *we define*

$$\bar{a}=\inf\left\{a:\int_{a}^{\infty }w\left(a\right)\;da=0\right\},\;\bar{b}=\inf\left\{b:\int_{b}^{\infty }\delta \left(b\right)\;db=0\right\}.$$

*Since the functions* *w*(*a*) *and* *δ*(*b*) *are in* $L_{+}^{1}\left(0,\infty \right)$, *we have* $\bar{a},\bar{b}>0$. *Additionally*, *let*

$$\tilde{X}=L_{+}^{1}\left(0,\infty \right)\times L_{+}^{1}\left(0,\infty \right)\times R^{+},\;\;\;\;\;\;\;\;\;\;\;\;\;\;\;\;\;\;\;\;\;\;\;\;\;\;\;\;\;\;\;\;\;\;\;\;\;\;\;\;\;\;\;\;\;\;\;\;\;\;\;\;\;\;\;\;\;\;\;\;\;\;\;\;\;\;\;\;\;\;\;\;\;$$

$$\tilde{Y}=\left\{{\left(v\left(t,\cdot \right),e\left(t,\cdot \right),I\left(t\right)\right)}^{T}\in \tilde{X}:\int_{0}^{\bar{a}}v\left(t,a\right)da>0\;\;or\int_{0}^{\bar{b}}e\left(t,b\right)db>0\;\;or\;\;I\left(t\right)>0\right\}.$$

*and define*

$$Y=R^{+}\times \tilde{Y},\;\partial Y=X\setminus Y,\;\partial \tilde{Y}=\tilde{X}\setminus \tilde{Y}.$$

Proposition 4.1 *Under the semiflow* {Φ(*t*)}_*t* ≥ 0_, *the subsets* *Y* *and* *∂Y* *are positively invariant*, *that is*, *for* *t* ≥ 0, Φ(*t*, *y*) ⊂ *Y* *and* Φ(*t*, *∂y*) ⊂ *∂Y*.

*Furthermore*, *the following theorem is useful for proving uniform persistence*.

Theorem 4.3 *For the semiflow* {Φ(*t*)}_*t* ≥ 0_ *restricted to* *∂Y*, *the disease-free equilibrium* *E*_0_ *is globally asymptotically stable*.

*Proof*: *Let* (*S*_0_, *v*(⋅), *e*(⋅), *I*_0_, *R*_0_, *M*_0_) ∈ *∂Y*, *then* $\left(v\left(t,\cdot \right),e\left(t,\cdot \right),I\left(t\right)\right)\in \partial \tilde{Y}$, *and we obtain the following system*:

$$\left\{\begin{array}{ll} & \frac{\partial v\left(t,a\right)}{\partial t}+\frac{\partial v\left(t,a\right)}{\partial a}=-\left(\sigma \left(a\right)\beta \left(M\left(t\right)\right)+w\left(a\right)+\mu \right)v\left(t,a\right),\\ & \frac{\partial e\left(t,b\right)}{\partial t}+\frac{\partial e\left(t,b\right)}{\partial b}=-\left(\delta \left(b\right)+\mu \right)e\left(t,b\right),\\ & \frac{dI\left(t\right)}{dt}=\int_{0}^{\infty }\delta \left(b\right)e\left(t,b\right)\;db-\left(\mu +\gamma +v\right)I\left(t\right),\\ & v\left(t,0\right)=p_{2}\mu A,\;e\left(t,0\right)=\beta \left(M\left(t\right)\right)S\left(t\right)I\left(t\right)+\int_{0}^{\infty }\sigma \left(a\right)\beta \left(M\left(t\right)\right)v\left(t,a\right)\;da,\\ & v\left(0,a\right)=v_{0}\left(a\right),\;e\left(0,b\right)=e_{0}\left(b\right),\;I\left(0\right)=0. \end{array}\right)$$

Since $S\left(t\right)+\int_{0}^{\infty }\sigma \left(a\right)\bar{v}\left(t,a\right)\;da\leq S\left(t\right)+\int_{0}^{\infty }\bar{v}\left(t,a\right)\;da\leq A$ *as* *t* → *∞*. *Additionally*,

(34) $$v\left(t,a\right)⩽\overset{⌣}{v}\left(t,a\right),\;\;\;\;\;\;\;\;e\left(t,b\right)⩽\overset{⌣}{e}\left(t,b\right),\;\;\;\;\;\;\;\;I\left(t\right)⩽\overset{⌣}{I}\left(t\right).$$

Here,

(35) $$\left\{\begin{array}{l} \frac{\partial \overset{⌣}{v}\left(t,a\right)}{\partial t}+\frac{\partial \overset{⌣}{v}\left(t,a\right)}{\partial a}=-\left(\sigma \left(a\right)\beta \left(M\left(t\right)\right)+w\left(a\right)+\mu \right)\overset{⌣}{v}\left(t,a\right),\\ \frac{\partial \overset{⌣}{e}\left(t,b\right)}{\partial t}+\frac{\partial \overset{⌣}{e}\left(t,b\right)}{\partial b}=-\left(\delta \left(b\right)+\mu \right)\overset{⌣}{e}\left(t,b\right),\\ \frac{d\overset{⌣}{I}\left(t\right)}{dt}=\int_{0}^{\infty }\delta \left(b\right)\overset{⌣}{e}\left(t,b\right)db-\left(\mu +\gamma +v\right)\overset{⌣}{I}\left(t\right),\\ \overset{⌣}{v}\left(t,0\right)=p_{2}\mu A,\overset{⌣}{e}\left(t,0\right)=A\int_{0}^{\infty }\overset{⌣}{I}\left(t\right)dt,\\ \overset{⌣}{v}\left(0,a\right)=v_{0}\left(a\right),\overset{⌣}{e}\left(0,b\right)=e_{0}\left(b\right),\overset{⌣}{I}\left(0\right)=0. \end{array}\right)$$

*Similar to* (5) *and* (6), *solving the second equation of* (35) *gives*

(36) $$\overset{⌣}{e}\left(t,b\right)=\left\{\begin{array}{ll} & A\int_{0}^{\infty }\overset{⌣}{I}\left(t-b\right)\rho_{2}\left(b\right)\;dt,\;t\geq b,\\ & e_{0}\left(b-t\right)\frac{\rho_{2}\left(b\right)}{\rho_{2}\left(b-t\right)},\;t<b. \end{array}\right)$$

*Substituting* (36) *into the third equation of* (35), *we obtain*

(37) $$\begin{array}{ll} \frac{d\overset{⌣}{I}\left(t\right)}{dt} & =\int_{0}^{t}\delta \left(b\right)A\int_{0}^{\infty }\overset{⌣}{I}\left(t-b\right)\rho_{2}\left(b\right)\;dt\;db+\int_{t}^{\infty }\delta \left(b\right)e_{0}\left(b-t\right)\frac{\rho_{2}\left(b\right)}{\rho_{2}\left(b-t\right)}\;db\\ & \;-\left(\mu +\gamma +v\right)\overset{⌣}{I}\left(t\right). \end{array}$$

*Denote the second term as* *G*(*t*). *Since* $\left(e_{0}\left(\cdot \right),I_{0}\right)\in \partial \tilde{Y}$, *then* *G*(*t*) ≡ 0 *for* *t* ≥ 0. *Therefore*,

(38) $$\left\{\begin{array}{ll} & \frac{d\overset{⌣}{I}\left(t\right)}{dt}=\int_{0}^{t}\delta \left(b\right)A\int_{0}^{\infty }\overset{⌣}{I}\left(t-b\right)\rho_{2}\left(b\right)\;dt\;db-\left(\mu +\gamma +v\right)\overset{⌣}{I}\left(t\right),\\ & \overset{⌣}{I}\left(0\right)=0. \end{array}\right)$$

*This has the unique solution* $\overset{⌣}{I}\left(t\right)=0$. *From* (36), *for* 0 ≤ *b* ≤ *t*, $\overset{⌣}{e}\left(t,b\right)=0$. *For* *b* > *t*, *we get*

$${\left\|\overset{⌣}{e}\left(t,b\right)\right\|}_{L^{1}}={\left\|e_{0}\left(b-t\right)\frac{\rho_{2}\left(b\right)}{\rho_{2}\left(b-t\right)}\right\|}_{L^{1}}\leq e^{-\left(\mu +\gamma +v\right)t}{\left\|e_{0}\right\|}_{L^{1}}.$$

*Thus*, $\lim_{t\to \infty }\overset{⌣}{e}\left(t,b\right)=0$. *From* (34), *we have* lim_*t*→*∞*_*e*(*t*, *b*) = 0 *and* lim_*t*→*∞*_*I*(*t*) = 0. *From the first and second equations of system* (1), *we derive* lim_*t*→*∞*_*S*(*t*) = *S*^0^ *and* lim_*t*→*∞*_*v*(*t*, *a*) = *v*^0^(*a*). *Therefore*, *the disease-free equilibrium* *E*_0_ *is globally asymptotically stable on* *∂Y*.

*Next*, *we prove that the semiflow* {Φ(*t*)}_*t* ≥ 0_ *is uniformly persistent on* *Y*.

Theorem 4.4 *Assume* *R*_0_ > 1. *The semiflow* {Φ(*t*)}_*t* ≥ 0_ *is uniformly persistent on* (*Y*, *∂Y*), *that is*, *there exists* *ɛ* > 0 (*independent of the initial values*) *such that for* *x* ∈ *Y* *and* *t* > *T*, lim inf_*t*→*∞*_‖Φ(*t*, *x*)‖_*X*_ ≥*ɛ*. *Moreover*, *the semiflow* {Φ(*t*)}_*t* ≥ 0_ *has a compact global attractor* $A_{0}$ *on* *Y*.

*Proof*: *By Theorem* 4.3, *E*_0_ *is globally asymptotically stable on* *∂Y*. *Therefore*, *it suffices to prove that there exist* *ɛ* > 0 (*independent of the initial values*) *and* *T* ≥ 0 *such that for any* *x* ∈ *Y* *and* *t* > *T*, lim inf_*t*→*∞*_‖Φ(*t*, *x*)‖_*X*_ ≥*ɛ*. *We only need to prove*

(39) $$W^{s}\left(E_{0}\right)\cap Y=\emptyset .$$

Here, $W^{s}\left(E_{0}\right)=\left\{x\in Y:\lim_{t\to \infty }\Phi \left(t,x\right)=E_{0}\right\}$.

*To avoid a contradiction*, *assume there exists a particular solution* *y* ∈ *Y* *such that* Φ(*t*, *y*) → *E*_0_ *as* *t* → *∞*. *It follows that we can find a sequence* {*y*_*n*_} ⊂ *Y* *such that for* *t* ≥ 0, $\|\Phi \left(t,y_{n}\right)-E_{0}{\|}_{X}<\frac{1}{n}$. *Here*, $\Phi \left(t,y_{n}\right)=\left(S_{n}\left(t\right),v_{n}\left(t,\cdot \right),e_{n}\left(t,\cdot \right),I_{n}\left(t\right),R_{n}\left(t\right),M_{n}\left(t\right)\right)$ *and* $y_{n}=\left(S_{n}\left(0\right),v_{n}\left(0,\cdot \right),e_{n}\left(0,\cdot \right),I_{n}\left(0\right),R_{n}\left(0\right),M_{n}\left(0\right)\right)$.

*Now*, *choose sufficiently large* *n* > 0 *such that* $S^{0}-\frac{1}{n}>0$ *and* $v^{0}\left(a\right)-\frac{1}{n}>0$. *For the given* *n* > 0, *there exists* *T* > 0 *such that for* *t* > *T*,

(40) $$S^{0}-\frac{1}{n}<S_{n}\left(t\right)<S^{0}+\frac{1}{n},\;v^{0}\left(a\right)-\frac{1}{n}<v_{n}\left(t,a\right)<v^{0}\left(a\right)+\frac{1}{n},\;0<I_{n}\left(t\right)<\frac{1}{n},\;0<M_{n}\left(t\right)<\frac{1}{n}.$$

*From the third equation of* (7), *we have*

(41) $$\begin{array}{ll} e\left(t,b\right) & =e\left(t-b,0\right)\rho_{2}\left(b\right)+e_{0}\left(b-t\right)\frac{\rho_{2}\left(b\right)}{\rho_{2}\left(b-t\right)}\\ & \geq \rho_{2}\left(b\right)e^{-\tau M}I\left(S\left(t-b\right)+\int_{0}^{\infty }\sigma \left(a\right)v\left(t-b,a\right)\;da\right). \end{array}$$

*By substituting* (40) *and* (41) *into the fourth equation of system* (1) *and applying the comparison principle*, *we obtain*

(42) $$I_{n}\left(t\right)\geq J_{n}\left(t\right),$$

*where* *J*_*n*_(*t*) *is the solution of the following system*:

$$\left\{\begin{array}{ll} \frac{dJ_{n}\left(t\right)}{dt} & =\int_{0}^{\infty }\delta \left(b\right)\rho_{2}\left(b\right)e^{-\tau M}I\left(\left(S^{0}-\frac{1}{n}\right)+\int_{0}^{\infty }\sigma \left(a\right)\left(v^{0}\left(a\right)-\frac{1}{n}\right)da\right)db\\ & \;-\left(\mu +\gamma +v\right)J_{n}\left(t\right),\\ J_{n}\left(0\right) & =I_{n}\left(0\right)\geq 0. \end{array}\right)$$

*Assuming* *J*_*n*_(0) = 0, *then* *J*_*n*_(*t*) ≥ 0. *Therefore*, *without loss of generality*, *we choose* *J*_*n*_(0) > 0. *Since* *R*_0_ > 1, *we choose sufficiently large* $n\in R^{+}$ *to ensure*

$$\int_{0}^{\infty }\delta \left(b\right)\rho_{2}\left(b\right)e^{-\tau M}I\left(\left(S^{0}-\frac{1}{n}\right)+\int_{0}^{\infty }\sigma \left(a\right)\left(v^{0}\left(a\right)-\frac{1}{n}\right)da\right)db>\left(\mu +\gamma +v\right).$$

*By* Lemma 4.1, *J*_*n*_(*t*) *is unbounded*. *Since* *I*_*n*_(*t*) ≥ *J*_*n*_(*t*), *I*_*n*_(*t*) *is also unbounded*. *Therefore*, Φ(*t*, *y*_*n*_) *is unbounded*, *which contradicts the boundedness of* *I*_*n*_(*t*). *Thus*, *W*^*s*^(*E*_0_) ∩ *Y* = ∅ *holds*. *By* (Li et al., 2010), {Φ(*t*)}_*t* ≥ 0_ *is uniformly persistent*, *and there exists a compact set* $A_{0}\subset Y$ *that is a global attractor for* {Φ(*t*)}_*t* ≥ 0_.

### 4.3 Global stability of steady-state solutions

Theorem 4.5 *When* *R*_0_ > 1, *the endemic equilibrium* *E*∗ *is globally asymptotically stable*.

*Proof*: *Construct the Lyapunov function*

$$V_{*}=W_{s}\left(t\right)+W_{v}\left(t\right)+W_{e}\left(t\right)+W_{i}\left(t\right)+W_{m}\left(t\right),$$

*where*

$$\begin{array}{l} W_{s}\left(t\right)=\rho_{1}S^{*}g\left(\frac{S}{S^{*}}\right),\;\;\;\;\;\;\;\;\;\;\;\;\;\;\;\;\;\;\;\;\;\;\;\;\;\;W_{v}\left(t\right)=\rho_{1}\int_{0}^{\infty }v^{*}\left(a\right)g\left(\frac{v\left(t,a\right)}{v^{*}\left(a\right)}\right)da,\\ W_{e}\left(t\right)=\int_{0}^{\infty }v_{2}\left(b\right)e^{*}\left(b\right)g\left(\frac{e\left(t,b\right)}{e^{*}\left(b\right)}\right)db,\;\;\;\;\;W_{i}\left(t\right)=I^{*}g\left(\frac{I}{I^{*}}\right),\\ W_{m}\left(t\right)=\frac{\rho_{1}\beta \left(M^{*}\right)S^{*}I^{*}}{u_{1}\alpha I^{*}}U\left(M\right). \end{array}$$

*here*, $U\left(M\right)=M-M^{*}-\int_{M^{*}}^{M}\frac{\beta \left(s\right)}{\beta \left(M^{*}\right)}ds$. *Since* $\mu =p_{1}\mu A\frac{1}{S^{*}}-\beta \left(M^{*}\right)+\frac{1}{S^{*}}\int_{0}^{\infty }w\left(a\right)v^{*}\left(a\right)da$, *then*

$$\begin{array}{ll} \frac{dW_{s}\left(t\right)}{dt} & =\rho_{1}\left(1-\frac{S^{*}}{S}\right)\\ & \;\left[p_{1}\mu A\left(1-\frac{S}{S^{*}}\right)+\beta \left(M^{*}\right)S^{*}I^{*}\left(\frac{S}{S^{*}}-\frac{\beta \left(M\right)SI}{\beta \left(M^{*}\right)}\right)S^{*}I^{*}+\int_{0}^{\infty }w\left(a\right)v^{*}\left(a\right)\left(\frac{v\left(t,a\right)}{v^{*}\left(a\right)}-\frac{S}{S^{*}}\right)da\right]\\ & =\rho_{1}p_{1}\mu A\left(2-\frac{S}{S^{*}}-\frac{S^{*}}{S}\right)+\rho_{1}\beta \left(M^{*}\right)S^{*}I^{*}\left(\frac{S}{S^{*}}-\frac{\beta \left(M\right)SI}{\beta \left(M^{*}\right)S^{*}I^{*}}-1+\frac{\beta \left(M\right)}{\beta \left(M^{*}\right)}\right)\\ & \;+\rho_{1}\int_{0}^{\infty }w\left(a\right)v^{*}\left(a\right)\left(\frac{v\left(t,a\right)}{v^{*}\left(a\right)}-\frac{S}{S^{*}}-\frac{S^{*}v\left(t,a\right)}{Sv^{*}\left(a\right)}+1\right)da. \end{array}$$

*The time derivative of* *W*_*v*_(*t*) *along the solutions of system* (1) *is*

$$\begin{array}{ll} \frac{dW_{v}\left(t\right)}{dt} & =\rho_{1}\int_{0}^{\infty }v^{*}\left(a\right)\frac{\partial }{\partial t}g\left(\frac{v\left(t,a\right)}{v^{*}\left(a\right)}\right)da\\ & =-\rho_{1}\int_{0}^{\infty }v^{*}\left(a\right)\left(1-\frac{v\left(t,a\right)}{v^{*}\left(a\right)}\right)\frac{1}{v^{*}\left(a\right)}\left[\frac{\partial }{\partial a}v\left(t,a\right)+\left(\sigma \left(a\right)\beta \left(M\right)+w\left(a\right)+\mu \right)v\left(t,a\right)\right]da\\ & =-\rho_{1}\int_{0}^{\infty }v^{*}\left(a\right)\left(\frac{v\left(t,a\right)}{v^{*}\left(a\right)}-1\right)\left(\frac{v_{a}\left(t,a\right)}{v\left(t,a\right)}+\left(\sigma \left(a\right)\beta \left(M^{*}\right)+w\left(a\right)+\mu \right)\right)da\\ & \;-\rho_{1}\int_{0}^{\infty }\sigma \left(a\right)v^{*}\left(a\right)\beta \left(M^{*}\right)\left(\frac{v\left(t,a\right)}{v^{*}\left(a\right)}-1\right)\left(\frac{\beta \left(M\right)}{\beta \left(M^{*}\right)}-1\right)da. \end{array}$$

*From* $\frac{dv^{*}\left(a\right)}{da}=-\left(\sigma \left(a\right)\beta \left(M^{*}\right)+w\left(a\right)+\mu \right)v^{*}\left(a\right)$, *we have*

$$\frac{\partial }{\partial a}g\left(\frac{v\left(t,a\right)}{v^{*}\left(a\right)}\right)=\left(\frac{v\left(t,a\right)}{v^{*}\left(a\right)}-1\right)\left(\frac{v_{a}\left(t,a\right)}{v\left(t,a\right)}+\left(\sigma \left(a\right)\beta \left(M^{*}\right)+w\left(a\right)+\mu \right)\right).$$

*Further*, *using integration by parts and* $g\left(\frac{v\left(t,0\right)}{v^{*}\left(0\right)}\right)=g\left(1\right)=0$, *we get*

$$\begin{array}{ll} \int_{0}^{\infty }v^{*}\left(a\right)\frac{\partial }{\partial a}g\left(\frac{v\left(t,a\right)}{v^{*}\left(a\right)}\right)da & ={\left(v^{*}\left(a\right)g\left(\frac{v\left(t,a\right)}{v^{*}\left(a\right)}\right)\right|}_{a=0}^{a=\infty }-\int_{0}^{\infty }g\left(\frac{v\left(t,a\right)}{v^{*}\left(a\right)}\right)\frac{dv^{*}\left(a\right)}{da}da\\ & =v^{*}\left(a\right){\left(g\left(\frac{v\left(t,a\right)}{v^{*}\left(a\right)}\right)\right|}_{a=\infty }+\int_{0}^{\infty }v^{*}\left(a\right)\left(\sigma \left(a\right)\beta \left(M^{*}\right)+w\left(a\right)+\mu \right)g\left(\frac{v\left(t,a\right)}{v^{*}\left(a\right)}\right)da. \end{array}$$

*Therefore*,

$$\begin{array}{ll} \frac{dW_{v}\left(t\right)}{dt} & =-\rho_{1}\int_{0}^{\infty }v^{*}\left(a\right)\frac{\partial }{\partial a}g\left(\frac{v\left(t,a\right)}{v^{*}\left(a\right)}\right)da-\rho_{1}\int_{0}^{\infty }\sigma \left(a\right)v^{*}\left(a\right)\beta \left(M^{*}\right)\left(\frac{v\left(t,a\right)}{v^{*}\left(a\right)}-1\right)\left(\frac{\beta \left(M\right)}{\beta \left(M^{*}\right)}-1\right)da\\ & =-\rho_{1}v^{*}\left(a\right){\left(g\left(\frac{v\left(t,a\right)}{v^{*}\left(a\right)}\right)\right|}_{a=\infty }-\rho_{1}\int_{0}^{\infty }v^{*}\left(a\right)\left[\sigma \left(a\right)\beta \left(M^{*}\right)+w\left(a\right)+\mu \right]g\left(\frac{v\left(t,a\right)}{v^{*}\left(a\right)}\right)da\\ & \;-\rho_{1}\int_{0}^{\infty }\sigma \left(a\right)v^{*}\left(a\right)\beta \left(M^{*}\right)\left(\frac{v\left(t,a\right)\beta \left(M\right)}{v^{*}\left(a\right)\beta \left(M^{*}\right)}-\frac{v\left(t,a\right)}{v^{*}\left(a\right)}-\frac{\beta \left(M\right)}{\beta \left(M^{*}\right)}+1\right)da\\ & =-\rho_{1}v^{*}\left(a\right){\left(g\left(\frac{v\left(t,a\right)}{v^{*}\left(a\right)}\right)\right|}_{a=\infty }-\rho_{1}\int_{0}^{\infty }v^{*}\left(a\right)\left(w\left(a\right)+\mu \right)g\left(\frac{v\left(t,a\right)}{v^{*}\left(a\right)}\right)da\\ & \;-\rho_{1}\int_{0}^{\infty }\sigma \left(a\right)v^{*}\left(a\right)\beta \left(M^{*}\right)\left(\frac{v\left(t,a\right)\beta \left(M\right)}{v^{*}\left(a\right)\beta \left(M^{*}\right)}-\frac{\beta \left(M\right)}{\beta \left(M^{*}\right)}-\ln\frac{v\left(t,a\right)}{v^{*}\left(a\right)}+1\right)da. \end{array}$$

*Similarly*, *the time derivative of* *W*_*e*_(*t*) *along the solutions of system* (1) *is*

$$\begin{array}{ll} \frac{dW_{e}\left(t\right)}{dt} & =\int_{0}^{\infty }v_{2}\left(b\right)e^{*}\left(b\right)\frac{\partial }{\partial t}g\left(\frac{e\left(t,b\right)}{e^{*}\left(b\right)}\right)db\\ & =-\int_{0}^{\infty }v_{2}\left(b\right)e^{*}\left(b\right)\left(1-\frac{e^{*}\left(b\right)}{e\left(t,b\right)}\right)\frac{1}{e^{*}\left(b\right)}\left(\frac{\partial }{\partial b}e\left(t,b\right)+\left(\delta \left(b\right)+\mu \right)e\left(t,b\right)\right)db\\ & =-\int_{0}^{\infty }e^{*}\left(b\right)\left(\frac{e\left(t,b\right)}{e^{*}\left(b\right)}-1\right)\left(\frac{e_{b}\left(t,b\right)}{e\left(t,b\right)}+\delta \left(b\right)+\mu \right)db. \end{array}$$

Here, $e_{b}\left(t,b\right)$ *denotes* $\frac{\partial e\left(t,b\right)}{\partial b}$.

*Since* $\frac{\partial }{\partial t}g\left(\frac{e\left(t,b\right)}{e^{*}\left(b\right)}\right)=\left(\frac{e\left(t,b\right)}{e^{*}\left(b\right)}-1\right)\left(\frac{e_{b}\left(t,b\right)}{e\left(t,b\right)}+\delta \left(b\right)+\mu \right)$, *then applying integration by parts and* $\frac{dv_{2}\left(b\right)}{db}=\delta \left(b\right)v_{2}\left(b\right)-\delta \left(b\right)$, *we obtain*

$$\begin{array}{ll} \frac{dW_{e}\left(t\right)}{dt} & =-\int_{0}^{\infty }v_{2}\left(b\right)e^{*}\left(b\right)\frac{\partial }{\partial b}g\left(\frac{e\left(t,b\right)}{e^{*}\left(b\right)}\right)db\\ & =-v_{2}\left(b\right)e^{*}\left(b\right){\left(g\left(\frac{e\left(t,b\right)}{e^{*}\left(b\right)}\right)\right|}_{b=0}^{b=\infty }+\int_{0}^{\infty }e^{*}\left(b\right)g\left(\frac{e\left(t,b\right)}{e^{*}\left(b\right)}\right)\frac{dv_{2}\left(b\right)}{db}db+\int_{0}^{\infty }v_{2}\left(b\right)g\left(\frac{e\left(t,b\right)}{e^{*}\left(b\right)}\right)\frac{de^{*}\left(b\right)}{db}db\\ & =-v_{2}\left(b\right)e^{*}\left(b\right){\left(g\left(\frac{e\left(t,b\right)}{e^{*}\left(b\right)}\right)\right|}_{b=\infty }+v_{2}\left(b\right)e^{*}\left(0\right)g\left(\frac{e\left(t,0\right)}{e^{*}\left(0\right)}\right)-\int_{0}^{\infty }\delta \left(b\right)e^{*}\left(b\right)g\left(\frac{e\left(t,b\right)}{e^{*}\left(b\right)}\right)db. \end{array}$$

*From the fourth equation in* (11), *we can deduce that* $\mu +\gamma +v=\frac{1}{I^{*}}\int_{0}^{\infty }\delta \left(b\right)e^{*}\left(b\right)db$, *then*

$$\begin{array}{ll} \frac{dW_{i}\left(t\right)}{dt} & =\left(1-\frac{I^{*}}{I}\right)\int_{0}^{\infty }\delta \left(b\right)e^{*}\left(b\right)\left(\frac{e\left(t,b\right)}{e^{*}\left(b\right)}-\frac{I}{I^{*}}\right)db\\ & =\int_{0}^{\infty }\delta \left(b\right)e^{*}\left(b\right)\left(\frac{e\left(t,b\right)}{e^{*}\left(b\right)}-\frac{I}{I^{*}}-\frac{e\left(t,b\right)I^{*}}{e^{*}\left(b\right)I}+1\right)db. \end{array}$$

$$\begin{array}{ll} \frac{dW_{m}\left(t\right)}{dt} & =\frac{\rho_{1}\beta \left(M^{*}\right)S^{*}I^{*}}{u_{1}\alpha I^{*}}\left(u_{1}\alpha I\left(t\right)-dM\left(t\right)-\frac{u_{1}\alpha \beta \left(M\right)I}{\beta \left(M^{*}\right)}+\frac{dM\beta \left(M\right)}{\beta \left(M^{*}\right)}\right)\\ & =\rho_{1}\beta \left(M^{*}\right)S^{*}I^{*}\left(\frac{I}{I^{*}}-\frac{dM}{\alpha I}-\frac{\beta \left(M\right)I}{\beta \left(M^{*}\right)I^{*}}+\frac{dM\beta \left(M\right)}{u_{1}\alpha \beta \left(M^{*}\right)M^{*}}\right). \end{array}$$

*Therefore*, *it can be shown that*

(45) $$\begin{array}{ll} \frac{dV_{*}\left(t\right)}{dt} & =\frac{dW_{s}\left(t\right)}{dt}+\frac{dW_{v}\left(t\right)}{dt}+\frac{dW_{e}\left(t\right)}{dt}+\frac{dW_{i}\left(t\right)}{dt}+\frac{dW_{m}\left(t\right)}{dt}\\ & =\rho_{1}S_{0}\left(1-\frac{S\left(t\right)}{S_{0}}\right)\left[p_{1}\mu A\left(1-\frac{S\left(t\right)}{S_{0}}\right)-\beta \left(M\right)S\left(t\right)I\left(t\right)+\int_{0}^{\infty }w\left(a\right)v\left(t,a\right)\;da\right]\\ & \;+\left(-\rho_{1}\int_{0}^{\infty }v_{0}\left(a\right)\left(1-\frac{v\left(t,a\right)}{v_{0}\left(a\right)}\right)\left[\frac{\partial }{\partial a}v\left(t,a\right)+\left(\sigma \left(a\right)\beta \left(M\right)+w\left(a\right)+\mu \right)v\left(t,a\right)\right]\;da\right)\\ & \;+\left(-\int_{0}^{\infty }v^{2}\left(b\right)e\left(t,b\right)\left(1-\frac{e\left(t,b\right)}{e^{*}\left(b\right)}\right)\left(\frac{\partial }{\partial b}e\left(t,b\right)+\left(\delta \left(b\right)+\mu \right)e\left(t,b\right)\right)\;db\right)\\ & \;+\left(\left(\int_{0}^{\infty }\delta \left(b\right)e\left(t,b\right)\;db-\left(\mu +\gamma +\nu \right)I\left(t\right)\right)\left(\frac{I\left(t\right)}{I^{*}}-1-\ln\left(\frac{I\left(t\right)}{I^{*}}\right)\right)+I\left(t\right)\left(\frac{1}{I^{*}}-\frac{1}{I\left(t\right)}\right)\right)\\ & \;+\left(u_{1}\alpha I\left(t\right)-dM\left(t\right)\right). \end{array}$$

*We can deduce that* $\frac{dV_{*}\left(t\right)}{dt}\leq 0$. *Since* *V*_∗_(*t*) *is bounded and non-increasing on* *ψ*(⋅), *this fact indicates that the* *α-limit set of* *ψ*(⋅) *must be contained in* Λ, *namely*, *the largest compact invariant subset where* $\left\{\frac{dV_{*}\left(t\right)}{dt}=0\right\}$. *A similar argument as in the* proof of Theorem 4.2 *shows that the* *α-limit of* *ψ*(⋅) *consists solely of the equilibrium point* *E*∗. *Furthermore*, *we have* *V*_∗_(*t*) ≥ *V*_∗_(*E*∗) *for all* t∈R. *Therefore*, *ψ*(*t*) ≡ *E*∗, *and then* $A_{0}=\left\{E^{*}\right\}$. *According to LaSalle’s Invariance Principle*, *the global asymptotic stability of* *E*∗ *in* **Y** *is established*, *completing the proof*.

*From* Theorem 4.1, Theorem 4.2, Theorem 4.5, *the following results can be derived*.

Theorem 4.6 *For the system* (1), *we have*

(i) *If* $R_{0}=\frac{\beta_{0}S_{0}\theta_{2}}{k}$, *then the DFE* *E*_0_ *is globally asymptotically stable*.
(ii) *If* *R*_0_ > 1, *then the unique EE* *E*∗ *is globally asymptotically stable*.

## 5 Numerical simulation

In this section, numerical simulations will be used to analyze and verify the aforementioned theoretical results.

First, the parameters are chosen as follows: *μ* = 0.0168, *τ* = 0.1, *p*_1_ = 0.03, *p*_2_ = 1 − *p*_1_ = 0.97, *A* = 100, *γ* = 0.02, *ν* = 0.002, *u*_1_ = 0.2, *α* = 0.05, *d* = 0.2. The initial conditions are *S*_0_ = 1000000, *v*_0_ = 10, *e*_0_ = 50, *I*_0_ = 100, *R*_0_ = 200, *M*_0_ = 10.

**Fig. 2:**
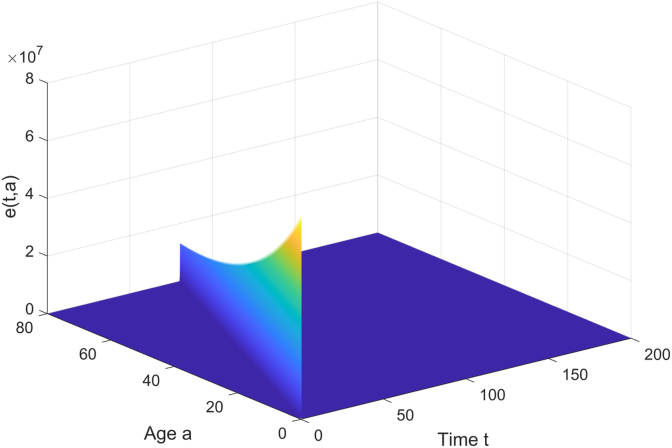
The curve of *E* changing with age and time.

**Fig. 3:**
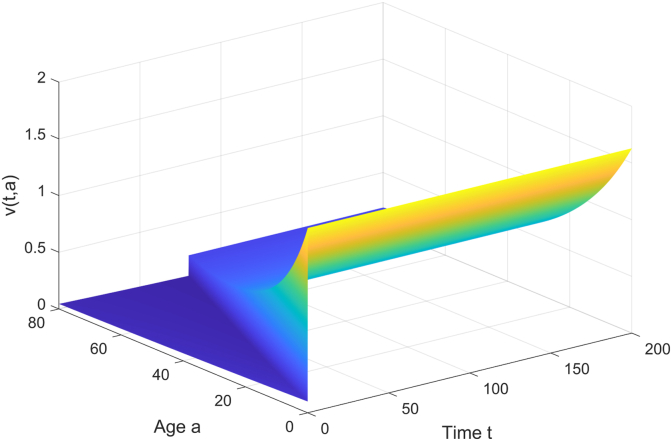
The curve of *V* changing with age and time.

The trends of the latent individuals *e*(*t*, *a*) and the vaccinated individuals *v*(*t*, *a*) over time *t* and age *a* are studied. As shown in Fig. 2, from the age dimension, the middle-aged and young adult groups (such as 18–50 years old) may have a higher proportion of latent individuals due to active social contact; the elderly group has fewer latent individuals due to lower contact rates. From the time dimension, the number of latent individuals rapidly increases at the early stage of the epidemic, and as media coverage and non-pharmaceutical interventions take effect, the infection rate decreases, and the number of latent individuals gradually reduces. Fig. 3 shows that due to the weak immunity of newborns, vaccination may prioritize covering newborns in hospitals, and the vaccination rate decreases with age and time, eventually stabilizing, with the lowest rate among the elderly due to their limited range of activities.

Next, the effective conversion rate *τ* from the susceptible compartment to the infected compartment, the influence parameter *u*_1_ of the infectives on the media, the recovery rate *γ* from the infected compartment to the recovered compartment, and the media dissipation rate *d* on *I*(*t*) and *M*(*t*) were studied respectively.

(1) The impact of the effective conversion rate ***τ*** from the susceptible compartment to the infected compartment

**Fig. 4:**
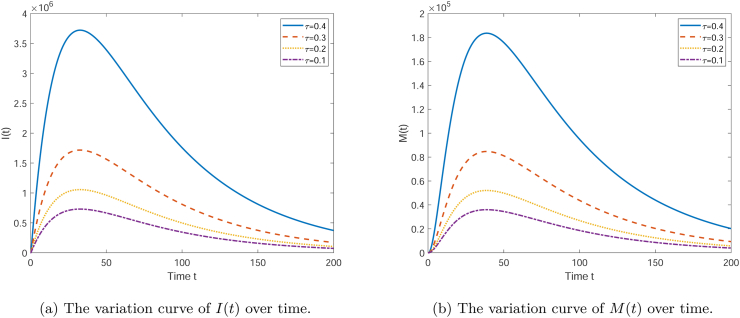
The curve graph showing the influence of *τ* on the quantities of each compartment.

Fig. 4 shows the curves of the numbers of *I*(*t*), *R*(*t*), and *M*(*t*) changing over time under the influence of different effective conversion rates *τ*. When *τ* takes *τ*, 2*τ*, 3*τ*, and 4*τ* respectively, it can be seen from Fig. 4(a) that as time *t* increases, the number of infectives *I*(*t*) first increases and then decreases, gradually approaching 0, presenting a peak. The curves with different *τ* values have different peak times and heights. A larger *τ* value (such as *τ* = 0.4) results in an earlier and higher peak, while a smaller *τ* value (such as *τ* = 0.1) causes the peak to appear later and lower. It can be seen from Fig. 4(b) that the media coverage *M*(*t*) also shows a trend of first increasing and then decreasing, but unlike *I*(*t*) and *R*(*t*), the variation in *M*(*t*) is greater, and the peak appears earlier. A larger *τ* value also leads to a higher peak for *M*(*t*). Overall, the parameter *τ* has a significant impact on these variables, with larger *τ* values usually resulting in faster dynamic changes and higher peaks. This may reflect the different response speeds and intensities of the system to external influences (such as media coverage) under different conditions.

(2) The influence of the parameter ***u*_1_**, which represents the impact of infectives on the media

**Fig. 5:**
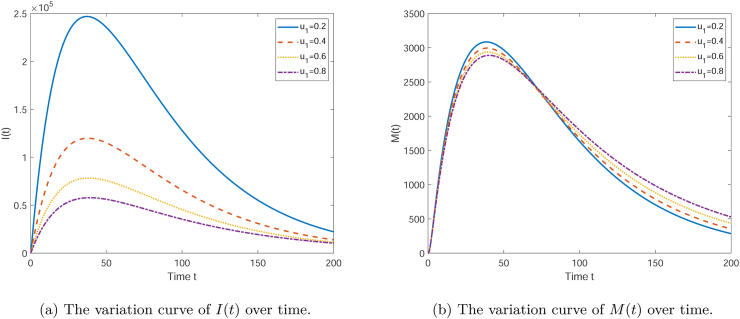
The variation curve graph showing the influence of *u*_1_ on the quantities of each compartment.

Fig. 5 illustrates the effect of the parameter *u*_1_ on the variables *I*(*t*) and *M*(*t*) over time. Each curve in the subplots corresponds to a different value of *u*_1_ (0.2, 0.4, 0.6, 0.8). As seen in Fig. 5(a), the number of infectives *I*(*t*) initially increases and then decreases over time, displaying a peak. With the increase of *u*_1_, the time at which the peak appears is earlier, and the peak height is reduced. This suggests that a higher *u*_1_ value may more effectively control the spread of infection. From Fig. 5(b), it can be observed that the media coverage *M*(*t*) also shows an initial increase followed by a decrease, but unlike *I*(*t*), the amplitude of change in *M*(*t*) is greater, and the peak appears earlier. A larger *u*_1_ value also results in a higher peak of *M*(*t*), which may reflect that under a higher *u*_1_ value, the media has a more significant impact on public awareness. Overall, an increase in the parameter *u*_1_ helps to achieve infection control more quickly and also enhances the media's impact on public awareness. This may suggest that in public health interventions, improving the efficiency and coverage of media campaigns is one of the effective strategies to control the spread of the epidemic.

(3) The influence of the recovery rate ***γ*** from the infected compartment to the recovered compartment

**Fig. 6:**
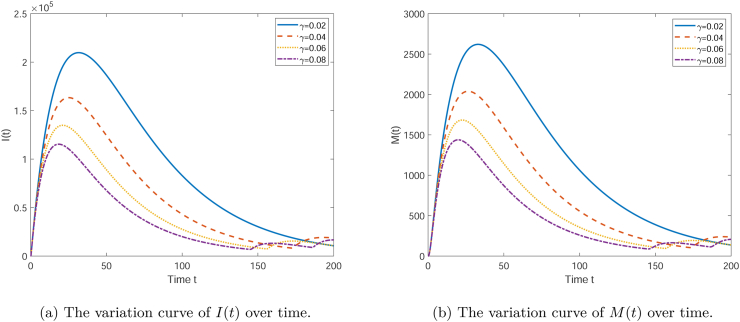
The variation curve graph showing the influence of *γ* on the quantities of each compartment.

Fig. 6 shows the effect of the parameter *γ* on the time-dependent changes of three different variables *I*(*t*) and *M*(*t*). Each subplot corresponds to one variable, with different values of *γ* (0.02, 0.04, 0.06, 0.08). As seen in Fig. 6(a), with the increase of *γ*, the peak of the number of infectives is reduced, and the time at which the peak appears is earlier. This indicates that a higher recovery rate helps to reduce the number of infectives more quickly. From Fig. 6(b), it can be seen how the media coverage *M*(*t*) changes over time. Similar to the previous two figures, a higher *γ* value results in a higher peak of *M*(*t*), which appears earlier. This may suggest that under a higher recovery rate, the media's impact is more significant, possibly because the increase in the recovery rate enhances public confidence in disease control. Overall, the parameter *γ* has a significant influence on the dynamic behavior of system. A higher *γ* value helps to control the spread of infection more quickly, increase the number of recoveries, and strengthen the media's impact. These results indicate that in public health interventions, increasing the recovery rate from diseases is one of the effective strategies to control the spread of the epidemic.

(4) The influence of the media dissipation rate ***d***

**Fig. 7:**
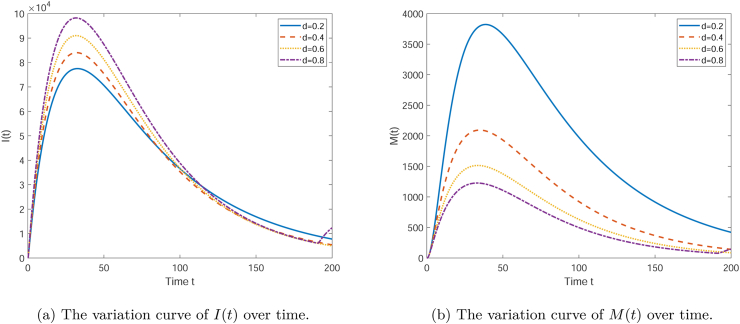
The variation curve graph showing the influence of *d* on the quantities of each compartment.

Fig. 7 illustrates the effect of the parameter *d* on the variables *I*(*t*) and *M*(*t*) over time. Each curve in the subplots corresponds to a different value of *d* (0.2, 0.4, 0.6, 0.8). As seen in Fig. 7(a), the number of infectives *I*(*t*) initially increases and then decreases over time, displaying a peak. With the increase of *d*, the peak of the infective population is reduced, and the time at which the peak appears is earlier. This indicates that a higher *d* value aids in reducing the number of infectives more quickly. From Fig. 7(b), it can be observed that the media coverage *M*(*t*) also shows an initial increase followed by a decrease, but unlike *I*(*t*), the amplitude of change in *M*(*t*) is greater, and the peak appears earlier. A larger *d* value also results in a higher peak for *M*(*t*), which may reflect a more significant impact of the media on public awareness under a higher *d* value. Overall, an increase in the parameter *d* helps to achieve infection control more rapidly and also enhances the media's impact on public awareness. This suggests that in public health interventions, increasing the mortality rate associated with the disease can effectively control the spread of the epidemic and may also enhance the effectiveness of media campaigns.

In summary, by effectively using the optimal control strategies for these parameters, the number of infections can be significantly reduced, the recovery process can be accelerated, and public awareness of the epidemic can be increased. In terms of the epidemic, as long as the prevention and control measures are well implemented, the overall situation can remain stable, and people can resume normal life as soon as possible. This emphasizes the importance of reasonably adjusting and controlling these parameters in public health interventions to achieve effective management and control of the epidemic.

## 6 Conclusion

This research focuses on the transmission characteristics of emerging infectious diseases under the influence of media information and age structure. An SVEIR-M age-structured infectious disease model is constructed, which comprehensively considers the immune effects of vaccination at different age stages, immune decay, heterogeneity of the incubation period, and the regulatory role of media information on the infection rate. By integrating epidemic transmission with information dissemination, the model authentically reflects the complex interactions between individual behavioral changes and epidemic transmission, theoretically revealing the synergistic reactions of combined interventions.

In the theoretical analysis section, the analysis first establishes the fundamental properties of the model, such as the existence and uniqueness of solutions. Subsequently, for the disease-free equilibrium and the endemic equilibrium of the system, the local and global stability of the equilibria are systematically analyzed by constructing appropriate Lyapunov functions and applying the Volterra integral method. When the basic reproduction number *R*_0_ < 1, the disease-free equilibrium is not only locally asymptotically stable but also globally attracts all non-negative solutions. When *R*_0_ > 1, the system exhibits uniform persistence and has a compact global attractor, ensuring the global asymptotic stability of the endemic equilibrium. These rigorous mathematical proofs provide a theoretical basis for understanding the transition from local outbreaks to sustained transmission of epidemics.

In numerical simulation experiments, the theoretical analysis results are further validated. The simulation results clearly show that increased media information can effectively reduce the infection rate, mainly due to its positive incentive effect on public protection awareness. Meanwhile, the promotion of vaccination significantly shortens the epidemic peak and reduces the number of infected individuals, with even more pronounced epidemic control effects when both are used in combination. The differences in the performance of individuals in different age groups during disease transmission are also well-reflected in the model, demonstrating the importance of age structure as a factor in modeling.

In summary, this study not only provides a new approach for constructing infectious disease models based on behavioral epidemiology but also offers theoretical support for the formulation of comprehensive intervention strategies. Future work could consider incorporating more refined contact networks, accounting for differences in information dissemination across different media platforms, and utilizing actual epidemiological data for parameter estimation and validation of the model, thereby further enhancing its application value in public health decision-making.

## CRediT authorship contribution statement

**Jianrong Wang:** Validation, Methodology, Investigation, Conceptualization. **Xue Yan:** Writing – original draft, Validation, Resources, Methodology. **Xinghua Chang:** Methodology, Investigation. **Maoxing Liu:** Writing – review & editing, Methodology, Investigation, Conceptualization.

## Declaration of interest

The authors declare that they have no known competing financial interests or personal relationships that could have appeared to influence the work reported in this paper.

## References

[bib1] Anderson R.M., May R.M. (1990).

[bib2] Bentout S., Tridane A., Djilali S., Touaoula T. (2021). Age-structured modeling of COVID-19 epidemic in the USA, UAE and Algeria. Alexandria Engineering Journal.

[bib3] Diagne M.L., Agusto F.B., Rwezaura H., Tchuenche J.M., Lenhart S. (2024). Optimal control of an epidemic model with treatment in the presence of media coverage. Scientific African.

[bib4] Du E., Chen E., Liu J., Zheng C.M. (2021). How do social media and individual behaviors affect epidemic transmission and control?. The Science of the Total Environment.

[bib7] Fome A.D., Rwezaura H., Diagne M.L., Collinson S., Tchuenche J.M. (2023). A deterministic SIR model for studying the impact of media on epidemic dynamics. Healthcare Analytics.

[bib11] Hao Y., Luo Y., Teng Z. (2025). Role of limited medical resources in an epidemic model with media report and general birth rate. Infectious Disease Modelling.

[bib12] Hethcote H.W. (2000). The mathematics of infectious diseases. SIAM Review.

[bib13] Huang J., Kang H., Lu M., Ruan S., Zhuo W. (2022). Stability analysis of an age-structured epidemic model with vaccination and standard incidence rate. Nonlinear Analysis: Real World Applications.

[bib14] Huo L., Yu Y. (2023). The impact of the self-recognition ability and physical quality on coupled negative information–behavior–epidemic dynamics in multiplex networks. Chaos, Solitons & Fractals.

[bib15] Khatua A., Kar T. (2020). Impacts of media awareness on a stage-structured epidemic model. Applied and Computational Mathematics.

[bib16] Kuniya T. (2019). Hopf bifurcation in an age-structured SIR epidemic model. Applied Mathematics Letters.

[bib17] Li X., Wang J., Ghosh M. (2010). Stability and bifurcation of an SIVS epidemic model with treatment and age of vaccination. Applied Mathematical Modelling.

[bib18] Li T., Xiao Y. (2022). Complex dynamics of an epidemic model with saturated media coverage and recovery. Nonlinear Dynamics.

[bib19] Li Y., Ye M., Zhang Q. (2019). Strong convergence of the partially truncated euler–Maruyama scheme for a stochastic age-structured SIR epidemic model. Applied Mathematics and Computation.

[bib20] Li H., Zhang L., Teng Z., Jiang Y., Muhammadhaji A. (2018). Global stability of an SI epidemic model with feedback controls in a patchy environment. Applied Mathematics and Computation.

[bib22] Liu R., Wu J., Zhu H. (2007). Media/psychological impact on multiple outbreaks of emerging infectious diseases. Computational and Mathematical Methods in Medicine.

[bib23] Luo Y., Liu P., Wu P., Teng Z. (2025). Dynamics of a nonlocal dispersal multi-group epidemic model with media-related nonlinear incidence in heterogeneous environment. Chaos, Solitons & Fractals.

[bib24] Meng R., Zheng T., Luo Y., Teng Z. (2025). Global attractor for an age-structured HIV model with nonlinear incidence rate. Applied Mathematics Letters.

[bib25] Omondi E., Imbusi N.M., Ananda K. (2024). The epidemiological impact of media campaigns on the dynamics of HIV transmission model. Research in Mathematics.

[bib26] Roy M., Moreau N., Rousseau C., Mercier A., Wilson A., Atlani L. (2020). Ebola and localized blame on social media: Analysis of Twitter and Facebook conversations during the 2014–2015 Ebola epidemic. Culture Medicine and Psychiatry.

[bib27] Sonveaux C., Joseph J. (2022). Design of a vaccination law for an age-dependent epidemic model using state feedback. IFAC-PapersOnLine.

[bib28] Sun D., Teng Z., Wang K., Zhang T. (2023). Stability and Hopf bifurcation in delayed age-structured SVIR epidemic model with vaccination and incubation. Chaos, Solitons & Fractals.

[bib29] Tchuenche J.M., Bauch C.T. (2012). Dynamics of an infectious disease where media coverage influences transmission. ISRN Biomathematics.

[bib30] Tchuenche J.M., Dube N., Bhunu C.P., Smith R.J., Bauch C.T. (2011). The impact of media coverage on the transmission dynamics of human influenza. BMC Public Health.

